# Understanding the Role of Morphology in the Visible-Light-Driven Sulfamethoxazole Degradation by Ag_2_SeO_3_‑Based Photocatalysts Synthesized in Different Solvent Media: An Experimental–Theoretical Approach

**DOI:** 10.1021/acs.inorgchem.5c04860

**Published:** 2026-01-21

**Authors:** Henrique Moreno, Laura O. Libero, Amanda Fernandes Gouveia, Marcio Daldin Teodoro, Monica Calatayud, Alexandre Zirpoli Simões, Elson Longo

**Affiliations:** † Center for Research and Development of Functional Materials, Federal University of São Carlos (UFSCar), 13565-905 São Carlos, Brazil; ‡ 27063 Sorbonne Université , CNRS, MONARIS, CNRS-UMR 8233, 4 Place Jussieu, F-75005 Paris, France; § Department of Physics, Federal University of São Carlos, São Carlos, SP 13565-905, Brazil; ∥ Faculty of Engineering of Guaratinguetá, São Paulo State University, 12516-410 Guaratinguetá, SP Brazil

## Abstract

The present research examines the photodegradation of sulfamethoxazole under visible light using Ag_2_SeO_3_ (ASO), synthesized via a sonochemical route in three different reaction environments: aqueous (ASOwat), ammoniacal (ASOamm), and ethanolic (ASOet) . X-ray diffraction, Raman, UV–visible, and photoluminescence spectroscopy measurements were carried out to further understand the structural changes driven by the synthesis̀ medium and their contribution to the photocatalysis. Additionally, field emission scanning electron microscopy revealed significant morphological differences among the samples, which were further confirmed by surface-energy calculations based on density functional theory associated with Wulff constructions. Our results indicate that surface-dependent band gap variations, influenced by the specific surface energy of the exposed facets, may assist in enabling partial visible-light activation, even though bulk ASO exhibits a larger band gap (*E*_gap_ ∼ 3.70 eV). The ASOet sample showed the highest performance (∼55% sulfamethoxazole degradation and 54% TOC mineralization) within 120 min. Thus, our work further reinforces the critical importance of particle morphology, which supersedes the overall band gap energy of the material, making visible-light excitation possible even for larger band gap materials.

## Graphical abstract

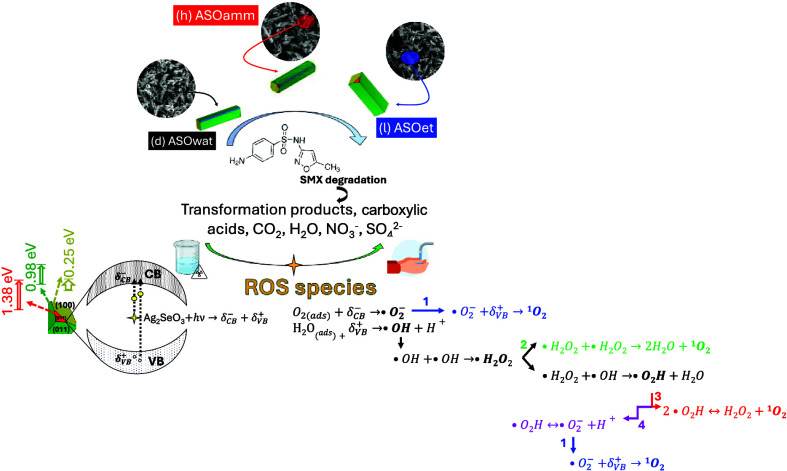

## 1 Introduction

In the past decade, the presence of recalcitrant contaminants in wastewater has become a trend worldwide.^1^ Among these, pharmaceutical compounds such as antibiotics, analgesics, and anti-inflammatories stand out due to their nature and difficulty of elimination.^2^ Despite their medicinal and agricultural importance, antibiotics have received considerable attention due to their pervasive presence, persistence, and ecological toxicity in aquatic ecosystems.^3–6^ These substances are classified as emerging contaminants because of their persistence and bioactivity in natural systems.^7^ According to projections by the World Health Organization, global antibiotic consumption in reported countries increased 16.3% from 29.5 to 34.3 billion defined daily doses (DDDs) from 2016 to 2023, reflecting a 10.6% increase in the consumption rate from 13.7 to 15.2 DDDs per 1000 inhabitants per day. It is anticipated to nearly quadruple between 2016 and 2030, reflecting an alarming upward trend.^8,9^ On the other hand, the United Nations (UN) proposed 17 Sustainable Development Goals (17 SDGs) to be achieved by 2030, through which it expects to slow down the environmental effects among several other goals.^1^

According to previous studies, over 200 pharmaceuticals have been reported in aquatic environments, which can be associated with several unwanted synergistic effects. Even the exposure to low concentrations of antibiotics can pose a significant threat to ecosystems and human health.^10^ Additionally, contamination of wastewater with pharmaceuticals is strongly connected to antibiotic-resistant bacteria and can even affect the removal of organic residues. Among the various antibiotics frequently identified in environmental monitoring studies, sulfamethoxazole (C_10_H_11_N_3_O_3_S, SMX) stands out as one of the most commonly detected compounds in surface and wastewater samples.^6,11^ SMX is a persistent organic pollutant having N-amine and carboxyl groups arising from the pharmaceutical industry. Upon consumption, it undergoes transformation reactions, thereby leading to the formation of oxidized, acetylated, and hydrolyzed metabolites in the environment. Though many countries banned SMX as a growth promoter for veterinary use, its occurrence in the environment is witnessed even today in rivers, lakes, groundwater, sediments, and the ocean. Photocatalysis arises as an alternative to conventional water treatment methods, which have proven ineffective for persistent organic contaminants.^12,13^

Among the various options, advanced oxidation processes (AOPs) have attracted a great deal of interest from researchers around the world because of their high degradation efficiency, even with large amounts of organic matter and low pH conditions, and their environmentally friendly nature. The study by Abellán et al.^14^ demonstrated that SMX photodegradation occurs through oxidation reactions, substitution processes, and cleavage of the double bond in the isoxazole ring. The authors reported the occurrence of hydroxylation (both mono- and polyhydroxylation), dealkylation, defluorination, and hydrolysis of the sulfonamide bond. According to Xie et al.,^15^ three main photodegradation mechanisms can be described: (1) radical attack at the N17 position of the sulfonamide group by hydroxyl radicals (^•^OH); (2) attack at the ortho positions (C_1_ and C_4_) of the aniline group by electrophilic radicals (*e.g.*, ^•^OH); and (3) attack at the N9 position of the aniline moiety, identified as one of the most vulnerable sites in SMX. Several studies have shown that local positive charge referred to as holes (h^+^), ^•^OH, and superoxide radicals (^•^O_2_^–^) are crucial for SMX degradation, with factors such as pH, ionic strength, and others being essential for optimizing the mineralization rate.^16–19^

Although AOPs have been widely investigated for the removal of pharmaceutical contaminants, the degradation of SMX remains challenging due to its chemical stability and low reactivity under conventional treatment. Most photocatalysts effective for SMX degradation rely on UV activation, limiting their applicability under visible light. This gap highlights the need for alternative semiconductor systems capable of operating under solar-relevant conditions. Generally, the efficiency of a photocatalyst depends on the ability of semiconductor materials to absorb light and on the separation, mobility, and recombination of charge carriers within the material.^20,21^ Wide band gaps are associated with light absorption in the ultraviolet region,^22,23^ limiting the application of semiconductor materials in photocatalytic processes. Ag_2_SeO_3_ (ASO) arises as a promising yet underexplored candidate due to its noncentrosymmetric structure and stereochemically active SeO_3_^2–^ lone pairs, which generate internal electric fields that can facilitate charge separation. Recent reports also suggest that its photocatalytic behavior is highly sensitive to surface termination and morphology, making it suitable for tuning through synthesis conditions.^24–26^

Apart from structural and electronic defects, this work provides conclusive evidence that the particle morphology is deterministic to the final properties of ASO. Theoretically, the intermediate band gap energy at ASO (*E*_gap_ ≈ 3.50 eV) favors UV light absorption. However, this work intends to show that the behavior of a semiconductor depends much more on its morphology, whose modulation can be used to tune surface properties depending on particle morphology distribution and surface energy, as well as on superficial cluster distribution. To the best of our knowledge, there is little information regarding the synthesis and photocatalytic properties of ASO via sonochemistry. This study presents a theoretical–experimental approach to evaluate the photodegradation of SMX under visible-light irradiation using ASO synthesized in different solvent media (water, ammonia, and ethanol). Our results contribute to elucidating the photodegradation of SMX, and, furthermore, highlight the importance of semiconductor morphology in catalytic processes.

## 2 Experimental Section

### 2.1 Synthesis

Ag_2_SeO_3_ was synthesized using the sonochemical method in three different reaction environments: (1) ASOwat—water, (2) ASOamm—2% NH_4_OH, and (3) ASOet—10% ethanol. AgNO_3_ (Synth, 99,0%) and SeO_2_ (Alfa Aesar, 99,4%) were used as starting reagents. The Se^4+^ precursor (1 × 10^–2^ mol) was sonicated in 50 mL of water for 15 min, while the Ag^+^ precursor was dissolved in deionized water (25 mL) using a vortex. The Ag^+^ solution (2 × 10^–2^ mol) was added dropwise to the Se^4+^ solution to form a white precipitate (ASO) and sonicated for another 30 min. Finally, the obtained suspension was kept in an ultrasonic bath (Branson, model 1510; frequency 42 kHz) (50 A) for 1 h at room temperature. The resulting powders were washed with deionized water, centrifuged 5 times, and then dried in an oven (60 °C) overnight. No uncommon hazards are noted.

### 2.2 Characterization

X-ray diffractometry (XRD) was conducted using a SmartLab SE (Rigaku, SP, Brazil) diffractometer operating at 40 kV and 60 mA, with Cu Kα radiation (λ = 1.5406 Å) over the 10–80° 2θ range and scanning rate of 0.2°/min to investigate phase formation of the samples. The XRD patterns were assessed by comparing ICSD (Inorganic Crystal Structure Database) standards, and Rietveld refinement of the structures was performed using the GSAS II software. Raman spectroscopy was conducted on a Horiba Jobin-Yvon (Japan) spectrometer charge-coupled device detector and an argon-ion laser (Melles Griot, SP, Brazil) operating at 633 nm with a maximum power of 17 mW. Fourier transform infrared spectroscopy (FTIR) was performed at room temperature using a Jasco FTIR-6200 (Japan) spectrophotometer operated in Attenuated Total Reflection (ATR) mode over the range of 400–4000 cm^–1^. Powder morphology was evaluated by using a field emission scanning electron microscope (FE-SEM, Zeiss Supra 35 at 5 kV, Germany). The band gap was estimated using the Kubelka–Munk function approach based on the diffuse reflectance spectra measurement.^27,28^ To do so, diffuse reflectance spectroscopy was carried out using a Shimadzu UV-1800 spectrophotometer (Japan) in diffuse reflectance (DRS) mode. Finally, photoluminescence spectroscopy was performed at room temperature using a 355 nm diode laser (Cobolt/Zouk) as an excitation source with a power of 1 mW at the sample.

X-ray photoelectron spectroscopy (XPS) was performed on a Scienta-Omicron ESCA+ equipped with a high-performance hemispheric analyzer (EA 125), with a monochromatic Al K_α_ X-ray source (300 W, *h*ν = 1486.6 eV), a high-performance hemispherical analyzer (EAC-2000), and a low-energy electron source to eliminate surface charging effects in insulating materials. The operating pressure in the ultrahigh vacuum chamber (UHV) during analysis was 2 × 10^–9^ mbar, and the data was recorded at a pass energy of 50 eV for survey scans and 30 eV for the high-resolution scans with a 0.5 and 0.05 eV, respectively. The XPS data was analyzed in the CasaXPS software (version 2.3.27PR4.4) using a Shirley-type background and Scoffield cross sections. All data were corrected to the C 1s peak for adventitious carbon, taken to be 284.8 eV, whose suitability was checked with the binding energy of the core-level peak binding energies. O 1s, Ag 3d, and Se 3p peaks were fitted using the GL(30) Voigt function in CasaXPS Software.

### 2.3 Theoretical Methods

The morphological evolution of the ASO crystals synthesized in different media was investigated through surface-energy calculations based on density functional theory (DFT) using the hybrid B3LYP exchange–correlation functional.^29,30^ Surface calculations were performed using the CRYSTAL17 program,^31,32^ following the methodology reported in the literature.^24^ Based on the FE-SEM images of the samples, the following low-index Miller surfaces were selected for investigation: (001), (010), (100), (011), (101), and (110). The (001) and (010) presented were modeled with two units of ASO, while the others contained three units. All surface models were constructed with a slab thickness of approximately 15 Å. A detailed structure analysis of the exposed atomic arrangements was carried out.

Surface-energy (*E*_surf_) values were computed using the conventional equation *E*_surf_ = 1/2A­(*E*_slab_–*nE*_bulk_), where *E*_slab_ is the total energy of the surface model, *E*_bulk_ is the energy per formula unit of the bulk, n is the number of formula units in the slab, and A is the surface area. Using these calculated *E*_surf_ and the Wulff construction, the equilibrium morphology of the ASO crystal was predicted, enabling a correlation between the synthesis environment and the observed morphological features. Additionally, the band gap energy (*E*_gap_) values of the surface models were calculated to assess their relationship with the photocatalytic performance. To gain further insight into the electron transfer mechanism at the surface, Mulliken charges and the density of states (DOS) of the atoms in the outermost layer were computed for each surface model.

### 2.4 Photocatalytic Analysis—SMX Degradation

The photocatalytic response was evaluated under visible-light irradiation with 6 lamps (Philips TL-D, 15 W, SP, Brazil). For each assay, 50 mg of the photocatalyst was suspended in 50 mL of a 1 ppm SMX solution using an ultrasonic bath (Branson, model 1510; frequency 42 kHz) for 5 min and then stirred in the dark for 30 min to reach absorption–adsorption equilibrium. Aliquots were collected when the lights were switched on (*t* = 0 min). The suspension was exposed to light under constant stirring under a controlled temperature of 20 °C, and aliquots were collected at specific times (*t* = 5, 10, 15, 20, 25, 30, 45, 60, 90, and 120 min), centrifuged to remove the supernatant, filtered using a syringe and 0.45 um filters, then monitored using high-performance liquid chromatography (HPLC). Experiments conducted in the dark (0, 5, 10, 15, 20, 25, 30, 45, 60, 90, and 120 min) were used to measure the contribution of absorption throughout the whole 120 min. Scavengers’ tests were used to indirectly identify the active species in the semiconductor and predict the degradation mechanism. The compounds used as scavengers were electrons (e^–^, silver nitrate), local positive charge (h^+^, ammonium oxalate), ^•^OH (potassium biftalate), and ^•^O_2_^–^ (ascorbic acid). Initial (0 min) and final (120 min) aliquots were withdrawn, centrifuged, and analyzed. The photocatalytic efficiency was calculated according to the percentage of absorbance of the dye solution using eq 1

1 $$\mathrm{degradation}\;\%=\frac{C_{0}-C_{t}}{C_{0}}$$

where *C*_0_ and *C*_*t*_ indicate the concentration of dye at time *t* = 0 and *t*, respectively.

To directly measure the formation of singlet oxygen (^1^O_2_) and hydroxyl (^•^OH) species during the photocatalytic reactions. For ^1^O_2_, 9,10-dimethylanthracene (DMA) (98%, Aldrich) probing was conducted using a UV–vis spectrophotometer (Jasco, Japan) in the 300–450 nm range, taking aliquots at specific times (0, 5, 10, 15, 20, 25, 30, 45, 60, 90, and 120 min). Analogously, coumarin (Aldrich) probing was carried out and analyzed in a spectrofluorophotometer (RF-5301 PC, Shimadzu, Japan) in the 250–350 nm range, taking aliquots at specific times (0, 5, 10, 15, 20, 25, 30, 45, 60, 90, and 120 min).

### 2.5 Chemical Analysis

The antibiotic concentration during the degradation process was monitored using HPLC, a Shimadzu Nexera X3 instrument equipped with a diode array detector (SPD-M40). A Shim-pack XR-ODS II analytical column (2.2 μm, 3.0 mm × 75 mm) was used, with the oven temperature set at 40 °C. The mobile phase consisted of 0.1% formic acid in ultrapure water/methanol (75:25), with isocratic elution at a flow rate of 0.4 mL min^–1^. The injection volume was 20 μL, and the detection was done at 270 nm. Under these conditions, SMX retention times were 1.45, and the quantification limit was 50 μg. L^–1^. The total organic carbon (TOC) was determined for each of the samples using a Shimadzu TOC-VCPN analyzer to determine the difference between inorganic carbon and organic carbon contents based on the NPOC method. The difference in IC obtained the TOC and TC measured values in generated CO_2_.^2,32^

## 3 Results and Discussion

### 3.1 Structural Characterization

Figure 1(a) shows the XRD diffractograms measured for the samples synthesized in different media. The data shows that, independently of the solvent mixture used, the phase ASO with the monoclinic symmetry and *P*2_1_/*c* space group was successfully obtained. The associated diffraction peaks were indexed according to the ICSD standard—*n*. 78388. Additional phase analysis was carried out using Rietveld refinement. The lattice and Rietveld parameters are shown in Figure S1­(a–c) and Table S1 of the Supporting Information (SI). Evaluation of the peak (032) shows a very slight increase in the full width at half-maximum (FWHM) values—0.18096, 0.17708, and 0.18138 for samples ASOwat, ASOamm, and ASOet, respectively—which can be suggestive of a more disordered lattice related to the synthesis in the ethanolic medium.

**Figure 1:**
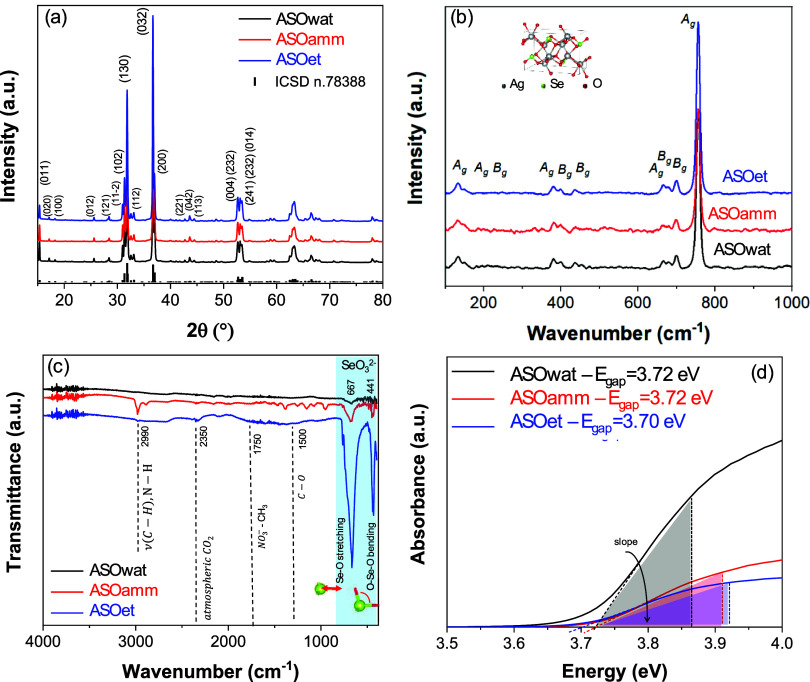
(a) XRD patterns, (b) Raman, (c) FTIR spectra, and (d) Kubelka–Munk Tauc plots obtained for all samples of the ASO system synthesized in different solvent environment conditions.

According to group theory analysis, the allowed representation for each of the corresponding Wyckoff positions of the structure of ASO in the symmetry group *D*_2h_ indicates 72 active modes in the Raman: Γ = 18A_g_ + 18A_u_ + 18B_g_ + 18B_u_. Raman spectroscopy provides critical insight into the local coordination and vibrational dynamics of ASO structures. The Raman spectra of the ASOwat, ASOamm, and ASOet samples (Figure 1(b)) all show prominent vibrational modes at approximately 133, 147, 382, 398, 435, 667, 679, 700, and 757 cm^–1^, which can be attributed to the distinct vibrational contributions of the SeO_3_^2–^ trigonal pyramidal units, Ag–O, and Ag–Se clusters. The lower wavenumber modes (133–147 cm^–1^) are commonly assigned to Ag–O and Ag–Se lattice vibrations and external modes involving Ag^+^ cations interacting weakly with oxygen or selenium ligands.^33^ Shifts at ∼133 cm^–1^ reflect Ag–Se bending or lattice vibrations, while the 147 cm^–1^ mode may also include Ag–O stretching.^34^ At 382–398 cm^–1^, the modes can be ascribed to Se–O bending in SeO_3_^2–^ groups. Such bending vibrations are sensitive to coordination asymmetry and reflect subtle differences in the SeO_3_^2–^ local environment across the different solvents.^35^ Additionally, the 435 cm^–1^ mode is also consistent with asymmetric bending or mixed vibrational modes of Se–O involving a higher degree of covalent character. Its relatively isolated presence may indicate a degree of intermediate-range ordering.

Furthermore, two modes at 667 and 700 cm^–1^ are characteristic of the asymmetric stretching of Se–O bonds within the SeO_3_^2–^ pyramidal units.^33,36^ The variation in the shape, intensity, and position among ASOwat, ASOamm, and ASOet suggests subtle changes in the Se–O bond lengths and angles, possibly arising from different solvent interactions during synthesis. Finally, the most intense mode identified is located at ∼757 cm^–1^ and can be assigned to the symmetric stretching vibration of the SeO_3_^2–^ group, which is typically Raman active due to the symmetry breaking in the distorted pyramidal geometry. The dominance of this mode across all samples supports the structural integrity of the SeO_3_^2–^ units under synthesis conditions.

In Raman spectroscopy, a larger FWHM is commonly interpreted as the result of increased structural disorder, defects, or local strain affecting the vibrational coherence of a unit, especially for stretching modes like ν_1_(Se–O).^37,38^ The main Raman mode *A*_*g*_ (∼757 cm^–1^) was also fitted, yielding a greater FWHM for sample ASOet (∼7.73 ± 0.07 cm^–1^) compared to ∼7.25 (±0.06) and ∼7.30 (±0.06) cm^–1^ for samples ASOwat and ASOamm, respectively. The broadening of this mode in the ASOet indicates a higher degree of short-range disorder. Thus, the ethanolic medium induces a more disordered structure, likely due to the interference of ethanol molecules in the nucleation and growth of ASO particles, which may have an influence on the electronic, optical, and catalytic properties of the material.

FTIR spectroscopy probes the vibrational modes of chemical bonds and functional groups, enabling the identification of molecular species and their interaction with the surrounding lattice. The FTIR spectra of the ASOwat, ASOamm, and ASOet samples (Figure 1(c)) exhibit prominent absorption bands at approximately 2990, 2350, 1392, 667, and 441 cm^–1^. These bands are consistent with residual solvent species, SeO_3_^2–^ unit vibrations, and lattice dynamics in the ASO systems. The broad absorption band at ∼2990 cm^–1^ can be assigned to C–H stretching vibrations of residual organic species. Its presence, especially in ASOet, is expected due to the organic content of the ethanol medium. This band is weak or absent in ASOwat, reflecting the more inorganic nature of the synthesis in water. Additionally, ammonium ions (NH_4_^+^) exhibit stretching bands typically in the 2800–3200 cm^–1^ range, depending on hydrogen bonding.^37^ The narrow nature of the band supports a less hydrogen-bonded, more free-state NH_4_^+^ or NH_3_ molecule interacting weakly with the ASO surface or trapped within its interstitial spaces. CO_2_ is also detected at ∼2350 cm^–1^. Finally, at ∼1392 cm^–1^, the broad band may originate from the CH_3_ bending of residual ethanol molecules. The band at ∼667 cm^–1^ is attributed to the asymmetric stretching vibration of Se–O bonds in the trigonal pyramidal SeO_3_^2–^ groups. It corresponds well with the Raman-active modes in the 667–700 cm^–1^ region and confirms the structural presence of SeO_3_^2–^ across all of the samples. The observed increase in intensity from ASOwat to ASOamm to ASOet indicates enhanced vibrational coupling or greater concentration/ordering of SeO_3_^2–^ groups in ASOet, potentially due to solvent medium effects on nucleation and growth dynamics. Finally, the band at ∼441 cm^–1^ is typically associated with the bending modes of Se–O bonds or Ag–O stretching vibrations in selenite frameworks. In some metal selenites, this region may also reflect Ag–Se bond deformation.^24,33,36^ The increasing intensity of this mode from ASOwat to ASOet suggests that ethanol promotes a structure where Ag–SeO_3_^2–^ bonding is more pronounced or better defined, possibly due to slower kinetics and more ordered growth under ethanolic conditions. Table 1 summarizes Raman and FTIR virbational modes detected for each of the samples.

**Table 1:** Raman and FTIR Vibrational Modes Observed for the ASO Lattice Synthesized in Different Solvent Environment Conditions

raman		FTIR	
mode	position (cm^–1^)	mode	position (cm^–1^)
Ag/Se–O	133	*ν*(C–H) stretching vibrations CO_2_ (*ν*)	2990
	147		
Se–O bending	382	asymmetric stretching vibration (*ν*_3_)	2350
	398		
Se–O bending	435	bending mode of NO_3_^–^ or deformation of CH_3_ groups	1392
Se–O asymmetric bending in SeO_3_^2–^(*ν*_3_)	667		
	679	ssymmetric stretching vibration (*ν*_3_) of Se–O	667
	700	Se–O bending or Ag–O/Ag–Se lattice modes	441
symmetric stretching in SeO_3_^2–^(*ν*_1_)	757		

The absorption dynamics of ASO microcrystals is characterized by allowed indirect electronic transitions between the valence band (VB) and the conduction band (CB). The optical band gap energy (*E*_gap_) for each of the samples was estimated based on diffuse reflectance measurements using the Kubelka–Munk^28^ function, based on the diffuse reflectance spectra (Figure 1(d)), which were 3.72 eV for samples ASOwat and ASOamm, slightly higher than that of sample ASOet (3.70 eV), similarly to what has been reported in the literature.^24^ Complementarily, Figure S2­(a–d) shows the absorption spectra and the derived Tauc plots for each of the samples and corroborating the estimated *E*_gap_ values. The absorption edges of all samples closely match the reflectance-derived band gaps, confirming that their optical response is dominated by intrinsic band-to-band transitions and that performance differences arise from surface-related effects rather than enhanced light absorption. Moreover, the similar absorption edges across the series demonstrate that bulk optical properties remain essentially unchanged. Therefore, the variations in photocatalytic performance among the samples are more plausibly linked to surface chemistry, defect density, and adsorption/charge-transfer dynamics, rather than to differences in light-harvesting capability. Corroborating the Raman data, an analysis of the Urbach tails^39^ (vertical in an ideal crystal) also indicates a more defective structure for the sample ASOet (lower slope) which can also be associated with distortions in bond angles in the [AgO_6_] and [SeO_3_] quantum clusters.

The theoretical *E*_gap_ values for all low-index Miller surfaces were calculated and are reported in Table 2, while the corresponding band structure plots are shown in Figure S3. Unlike the experimental data, and with the exception of the (100) surface, which exhibits a direct transition (Γ–Γ), all surfaces present an indirect transition between the VB and CB, typically involving the B and Γ–points, as illustrated in Figure S3. The (101) surface displays the lowest *E*_gap_ value, whereas the (001), (010), and (100) surfaces have values approximately close to the experimental band gap of 3.70 eV. These reduced *E*_gap_ values arise from the creation of intermediate electronic stated between the VB and CB. It is important to note that the band gap value depends on the surface termination,^40^ which explains the lower value obtained here compared to the data reported in the literature.^24^ Moreover, the calculated *E*_gap_ values for the different surfaces differ from those estimated by using UV–visible spectroscopy and the Kubelka–Munk approach. These discrepancies are significant, as they directly influence the reactivity of the semiconductor toward its environment and, consequently, its ability to generate ROS.

**Table 2:** Surface Energies (*E*_surf_, J/m^2^), Band Gap Energy (*E*_gap_, eV), Density of Broken Bonds (*D*_b_, nm^–2^), Relative Increase in *D*_b_ Compared to the Minimum Value (Δ*D*_b_, %), Mulliken Charges (e^–^) of the Clusters, and Percentage Contribution (%) of Each Exposed Surface in the Three Morphologies

surface	*E*_surf_	*E*_gap_	*D*_b_	Δ*D*_b_ (%)	mulliken charges of the clusters						ASOamm	ASOet	ASOwat
(001)	0.53	3.64	9.53	20.45	[AgO_5_]	[AgO_5_]	[AgO_3_]	[SeO_3_]	[SeO_3_]	[SeO_3_]	0.00	0.00	4.24
					–0.871	–0.630	–0.085	0.169	0.378	0.350			
(010)	0.52	3.78	8.52	7.64	[AgO_4_]	[AgO_5_]	[AgO_6_]	[SeO_3_]			6.47	0.00	4.11
					–0.662	–0.596	–1.058	0.148					
(100)	0.25	3.79	7.99	7.99	[AgO_5_]	[AgO_5_]	[AgO_4_]	[AgO_4_]	[SeO_3_]	[SeO_3_]	10.68	9.14	9.39
					–0.283	–0.283	–0.141	–0.141	0.509	0.509			
(011)	0.98	2.48	7.91	0.00	[AgO_4_]	[AgO_4_]	[AgO_5_]	[AgO_6_]	[SeO_3_]	[SeO_3_]	82.85	89.27	80.72
					–0.294	–0.400	–0.585	–0.788	0.558	0.277			
(101)	1.38	1.44	9.77	23.50	[AgO_3_]	[AgO_3_]	[AgO_3_]	[SeO_3_]	[SeO_3_]		0.00	1.60	0.37
					0.055	–0.063	–0.215	–1.333	0.433				
(110)	0.74	1.74	8.44	6.64	[AgO_3_]	[AgO_4_]	[AgO_3_]	[SeO_3_]	[SeO_3_]		0.00	0.00	1.16
					0.052	–0.092	–0.003	0.419	0.381				

Although the overall band gap for all samples was experimentally estimated to be ∼3.70 eV, each particle can be decomposed into distinct surfaces with different local surface energies and band gaps. For instance, the (101) surface shows the higher reactivity (*E*_surf_ = 1.38 eV; *E*_gap_ = 1.44 eV), while the surface (010) (*E*_surf_ = 0.52 eV; *E*_gap_ = 3.78 eV) and (100) (*E*_surf_ = 0.25 eV; *E*_gap_ = 3.79 eV) are comparatively more stable and less reactive.

To investigate the charge distribution at each surface termination, Mulliken charges were calculated for the clusters located in the outermost layer, and the results are also presented in Table 2. According to these values, less negative or even positive charges are observed as the number of oxygen atoms coordinating the cation decreases, indicating a loss of electron density, which is consistent with the presence of oxygen vacancies. On the (101) surface, the [SeO_3_] cluster exhibits a Mulliken charge with a high absolute value, suggesting the presence of deep defects associated with elongated Se–O bonds. In contrast, the (011), (100), and (010) surfaces display very similar charge values, indicating a comparable electronic behavior.

To further analyze the effect of electronic density on the atoms in the outermost Ag and Se cluster, the projected DOS was computed for each surface model, as shown in Figure S4. Several insights can be drawn. First, the decrease in the *E*_gap_ values is mainly due to contributions from [SeO_*x*_] clusters at the bottom of the CB. Surfaces with the lowest *E*_surf_, such as (100), exhibit homogeneous electronic behavior, both in their DOS and in Mulliken charges.

By contrast, in surfaces with lower *E*_gap_ values, the *V*_O_ induces a loss of degeneracy, and clusters with the same coordination number, such as the [AgO_3_] and [SeO_3_] on the (101) surface (Figure S4­(i,j) respectively), begin to show distinct DOS profiles and different Mulliken charges.

### 3.2 Morphology

Figure 2(a–i) exhibits the FE-SEM micrograph images obtained for the ASO samples. In addition to long and short-range order structure, the solvent environment plays a key role in the morphology of ASO microcrystals synthesized via sonochemistry. Moreover, the sonochemical method consists of an ultrasonic-assisted coprecipitation, and can be described by self-aggregation, Ostwald ripening (OR), and self-organization. The presence of NH_4_OH or ethanol (C_2_H_5_OH) during the synthesis induces an initial complexation of the Ag^+^ ions by these molecules. Due to steric effects, the number of effective shocks produced during sonication varies and influences the final morphology of the particles.

**Figure 2:**
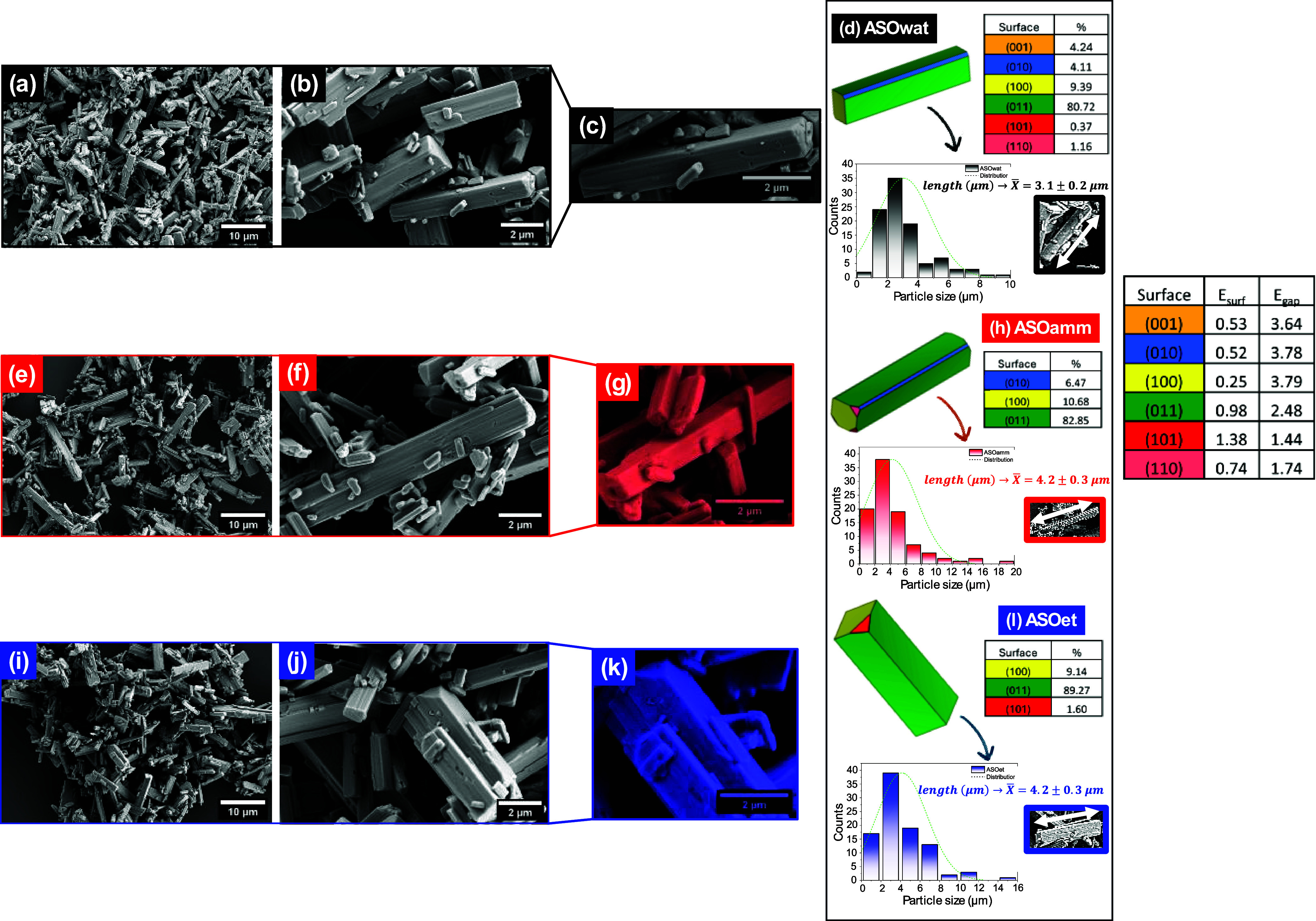
FE-SEM micrograph images obtained for the samples synthesized in (a–c) aqueous (ASOwat), (e–g) ammoniacal (ASOamm), and (i–k) ethanolic (ASOet) environments. The image shows 5k (a, e, i), 25k (b, f, j), and 40k (c, g, k) magnification micrograph images for each of the samples. The corresponding Wulff constructions are also shown (d, h, l), along with the associated *E*_surf_ and the percentage of contribution (%) of each exposed surface to the morphology. The surfaces are identified as follows: (001) in orange, (010) in blue, (100) in yellow, (011) in green, (101) in red, and (110) in pink. The inserts show the associated particle size distribution measured for each of the samples with the respective average particle size values.

The images revealed that the sample synthesized in water (ASOwat) exhibited loosely packed, anisotropic rod-like crystals with irregular surfaces, indicative of rapid nucleation and less controlled growth, a behavior consistent with the high polarity and fast ion transport of aqueous media. In contrast, the use of a 2% ammoniacal solution (ASOamm) led to more faceted and elongated microcrystals, with visible platelet-like surface features, suggesting moderated nucleation and oriented growth likely induced by complexation between Ag^+^ and NH_3_, which tend to form the [Ag­(NH_3_)_2_]^+^ complex, decreasing supersaturation and slowing crystal growth, while the lower dielectric constant and reduced ion diffusion in ethanol favor growth-dominated crystallization. Under sonication, differences in cavitation behavior further amplify these effects, leading to facet-selective stabilization and directional crystal development, confirming that anisotropic morphology is not solvent-random but driven by the interplay between surface energetics, solvent coordination, and sonochemical kinetics.

The most well-defined morphology was observed for the sample synthesized in 10% ethanol (ASOet), which displayed highly faceted monodisperse rods with increased surface modulation. This reflects the slower ion diffusion and selective surface adsorption properties of ethanol, likely due to the reduced dielectric constant and lower ion mobility in ethanol, which favor crystal growth over nucleation. The inserts in Figure 2 show the respective particle size distribution and average particle size for each of the samples.

Raman spectra showed a consistent Se–O symmetric stretching mode at ∼757 cm^–1^, but with increasing full width at half-maximum (FWHM) from ASOwat to ASOet, indicating greater local structural disorder in ethanol-derived samples. FTIR data corroborated this with Se–O asymmetric stretching (667 cm^–1^) and bending (441 cm^–1^) modes intensifying from ASOwat to ASOet, suggesting enhanced vibrational activity and improved short-range bonding definition in less polar media. Notably, a sharp and intense band at ∼2990 cm^–1^ in ASOamm was attributed to N–H stretching from residual ammonium species, while a broader, weaker band in ASOet was assigned to C–H stretching from ethanol residues. Although Raman and FTIR spectroscopy do not directly determine particle morphology, the solvent-dependent variations in band intensity and FWHM—particularly the broadening of the *ν*_1_(Se–O) mode in ASOet—indicate differences in local structural disorder and bonding environments that are consistent with the distinct nucleation and growth conditions leading to the observed morphologies

The directional growth of Ag_2_SeO_3_ microcrystals arises from a solvent-dependent modulation of the surface energetics and growth kinetics. DFT-derived Wulff constructions reveal that ethanol selectively stabilizes the high-energy (101) surface, whereas water and ammoniacal media favor exposure of the (010) and (100) planes. This shift in the surface-energy hierarchy promotes anisotropic growth along the [101] direction, resulting in the highly faceted rod-like particles observed for ASOet. Because crystal growth proceeds fastest on surfaces with higher residual surface energy, the ethanol-induced stabilization of the (101) facet reverses the relative surface-energy order, leaving this plane as the least passivated and therefore the most kinetically active. This imbalance drives anisotropic growth along the [101] axis, producing elongated, highly faceted, rod-like morphologies in the ASOet sample. Conversely, in aqueous and ammoniacal media, the more isotropic stabilization of the (010)/(100) facets results in compact, less anisotropic morphologies. To support the SEM analysis, the theoretical morphology of the ASO synthesized in aqueous, ammoniacal, and ethanolic media was also assessed, and is displayed in Figure 2(d,h,l). All samples present are dominated by the surfaces (011) (green). Additionally, one must account for the contribution of other surfaces at minor extents: (001), (010), (100), (101), and (110) in ASOwat; (010) and (100) in ASOamm; and (100) and (101) in sample ASOet. Therefore, the difference between ASOet and ASOamm lies in the substitution of the (101) surface in ASOet by the (010) surface in ASOamm. In the case of ASOwat, all of these surfaces are present, along with the additional exposure of the (001) surface.

To better understand the morphological characteristics of the ASO samples, the contribution of each exposed surface (%) to the overall crystal morphology was analyzed along with the density of broken bonds (*D*_b_) and the relative increase in *D*_b_ compared to the minimum value (Δ*D*_b_, %). The corresponding values are also presented in Table 2. This analysis is important for assessing the degree of order and disorder associated with each exposed surface.

All surfaces show a distribution among [AgO_*x*_] (AgO_3_, AgO_4_, AgO_5_, and AgO_6_) and [SeO_3_] quantum clusters. The exposed quantum clusters have different associated energies, which depend on the atomic distribution. The major surface (011) has a balance between negatively charged silver (AgO_4_, AgO_5_, and AgO_6_) and positively charged [SeO_3_] quantum clusters, producing a more stable environment with lower defect density (7.91 nm^–2^) compared to the other surfaces. The surfaces (010) and (100) yield total negative ([AgO_*x*_] clusters) and positive ([SeO_3_] clusters) charges, respectively. These results suggest that samples ASOwat and ASOamm behave as electron donors predominantly. On the other hand, in the sample, the ASOet surface (101), dominated by negatively charged silver and selenium quantum clusters, rises. Furthermore, the surface (101) possesses a higher density of broken bonds (13.03 nm^–2^)—approximately 64.7%— compared to the other surfaces ((011), 7.91 nm^–2^ and (100), 7.99 nm^–2^), which can be indicative of a lower oxygen vacancy density and a higher concentration of structural defects.

Figure 3(a–f) shows the deconvoluted high-resolution Ag 3d and O 1s XPS spectra obtained for each of the samples. The Ag 3d spectra (Figure 3(a,c,e)) obtained over the 377.5–365 eV range display two distinct well-defined doublets at ∼373 eV (Ag 3d_3/2_) and ∼367 eV (Ag 3d_5/2_), with a spin–orbit splitting of ∼6 eV. Deconvolution of these peaks reveals the coexistence of Ag^+^ and metallic Ag^0^. The Ag^+^ components (3d_5/2_ at ∼367.5 eV and 3d_3/2_ at ∼373.5 eV) confirm the formation of silver quantum clusters—Ag–O—within the Ag_2_SeO_3_ structure. In contrast, the Ag^0^ components (3d_5/2_ at ∼368.4 eV and 3d_3/2_ at ∼374.4 eV) can be ascribed to metallic silver species, which can be interpreted as surface-localized rather than bulk structural defects.^41,42^ Surface Ag^0^ may arise from partial reduction associated with beam-induced processes, as previously reported in the literature for silver-based semiconductors.^41,43^ Moreover, Figure S5­(a–c) displays the Se 3p high-resolution spectra for each of the samples. The Se 3p spectra show two peaks associated with the 3p spin–orbit coupling at 164 eV (Se 3p_3/2_) and 170 eV (Se 3p_1/2_), which can be ascribed to Se^4+^ species.^24^

**Figure 3:**
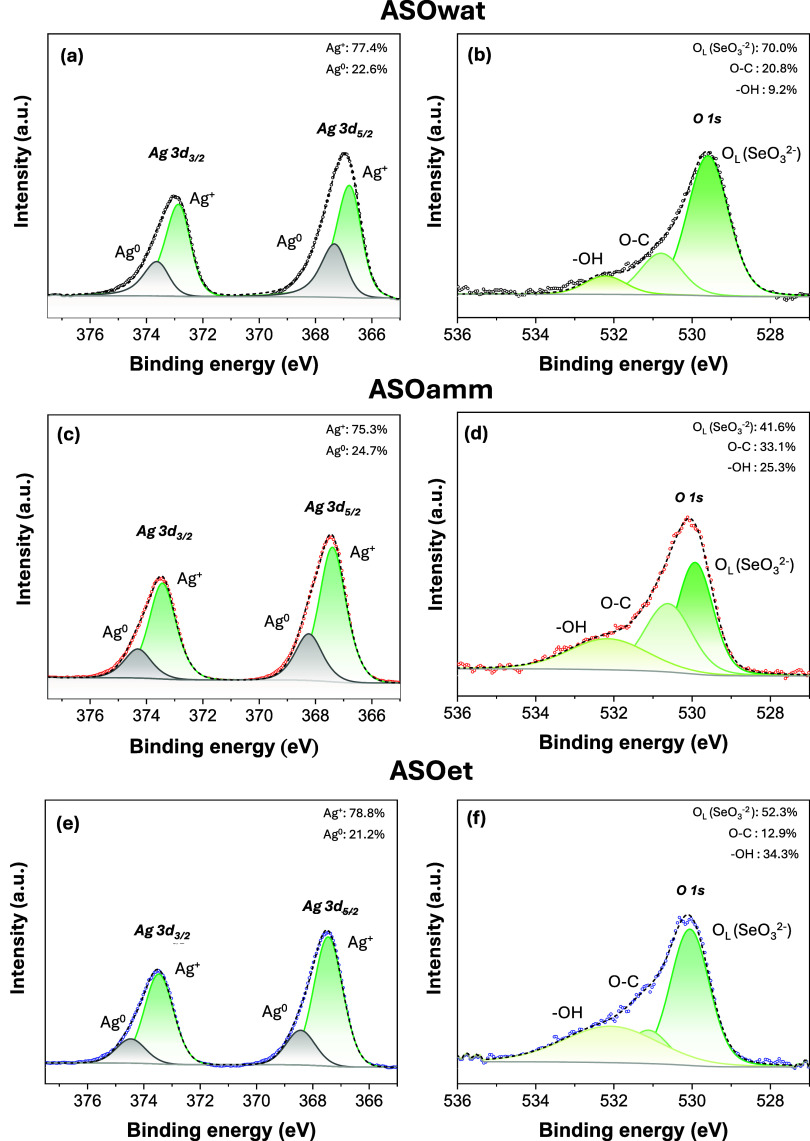
Ag 3d and O 1s high-resolution XPS spectra obtained for the samples synthesized in (a–b) aqueous (ASOwat), (c–d) ammoniacal (ASOamm), and (e–f) ethanolic (ASOet) environments.

The O 1s spectra, recorded in the 540–527 eV range, were fitted into three main contributions associated with lattice oxygen (O_L_) at ∼530.6 eV and the SeO_3_^2–^ bonds within the Ag_2_SeO_3_. At ∼ 531.7 eV, the peak can be ascribed to nonlattice oxygen bonds, possibly surface carbonyl/carboxylate oxygen from adsorbed adventitious carbon and/or residual organic species from synthesis. The coexistence of metallic Ag^0^ and Ag^+^ species in the Ag 3d spectra indicates a partial reduction of Ag, which can enhance the adsorption of carbonaceous species at the surface. The assignment of the ∼531.7 eV component to organics is further supported by (i) concomitant C 1s features at ∼285.8 eV (C–O) and 287.8–290.7 eV (O–C=O). Finally, surface hydroxyl groups and/or adsorbed water (and or residual ethanol) can be detected at ∼533.6 eV. Notably, the amount of oxygen species related to the oxide (SeO_3_^2–^) decreases when changing the synthesis medium, which may reflect an increase in the density of structural defects with the Ag_2_SeO_3_ lattice. On the other hand, the data signals a higher density of −OH bonds, signaling the presence of residual NH_4_OH/ethanol over the surface of the material. Altogether, these results confirm the coexistence of Ag in different oxidation states and suggest an increase in structural defects, which was shown to influence surface reactivity.

### 3.3 Optical Response

Figure 4(a) displays that the PL emission spectra for the ASO samples reveal significant solvent-dependent differences in defect-related emission behavior, directly tied to the electronic structure and defect chemistry of the material. All samples exhibit broad emissions in the visible to near-infrared region, consistent with recombination via intrinsic defect states rather than direct band-to-band transitions.^24,26^ Deconvolution of the spectra (Figure 4(b–d)) shows that ASOwat features a distribution of orange (∼2.0 eV), red (∼1.75 eV), and NIR (∼1.5 eV) emissions, with the red component being dominant. This pattern indicates the presence of both shallow- and deep-level defects (i.e., *V*_O_). These point defects are commonly introduced by the rapid nucleation and disorder associated with aqueous sonochemical conditions. In ASOamm, similar red-dominant behavior is observed but with a slightly increased contribution from deep NIR emission, suggesting enhanced incorporation of donor–acceptor pair defects, likely promoted by the partial complexation of Ag^+^ by NH_3_ during synthesis. The complexation of the Ag^+^ particles by ethanolic groups may increase the number of effective shocks during ASO synthesis in ASOet, which exhibits a highly uniform red emission (94%) with suppressed contributions from deeper traps, implying a lower *V*_O_ concentration in a defective structure environment. These PL trends have direct implications for photocatalytic behavior, corroborating both theoretical and XPS data.

**Figure 4:**
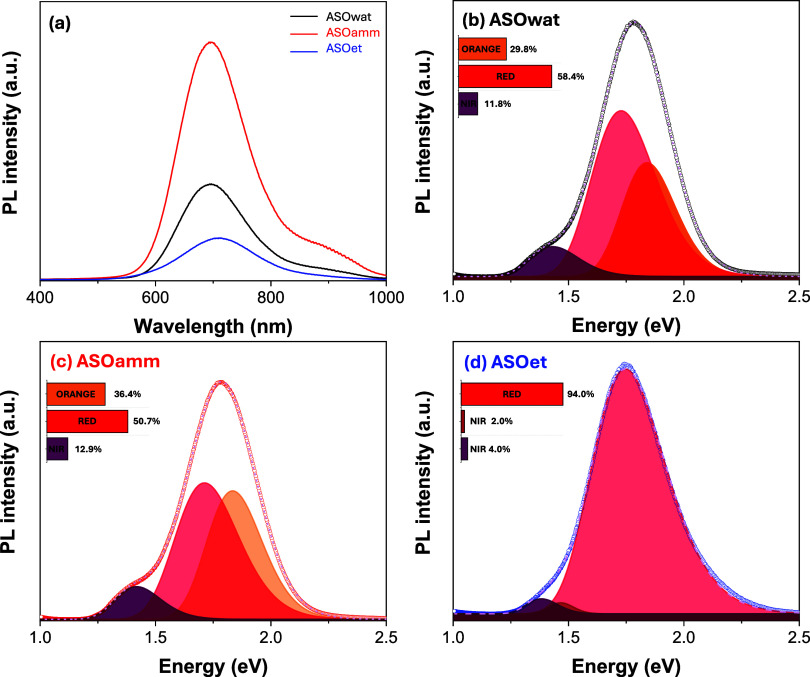
(a) PL spectra (λ_ex_ = 633 nm) and the related deconvolution for the samples synthesized in (b) aqueous (ASOwat), (c) ammoniacal (ASOamm), and (d) ethanolic (ASOet) environments.

Although PL does not directly measure charge-carrier dynamics, lower deep-level emission intensity is consistent with suppressed nonradiative recombination and more efficient charge separation.^44,45^ This behavior agrees with the superior photocatalytic performance of ASOet, whereas ASOwat and ASOamm, which display broader and more intense deep-level PL components, exhibit lower degradation efficiencies.

Optimized charge-carrier separation and surface reactivity may stem from the formation of structural defects at the expense of shallow-level defects and traps associated with *V*_O_^*•*^. A joint evaluation of the disorder-defect state in the material suggests that the broader *ν*_1_(Se–O) Raman mode and increased Urbach tail in ASOet reflect higher short-range lattice distortion, which is likely associated with local strain and coordination asymmetry rather than higher oxygen vacancy density. In contrast, PL deconvolution shows a reduced contribution from deep-level emissions, indicating fewer recombination-active defects.

To evaluate the structural integrity and the morphology of the ASO particles under visible-light-driven SMX degradation, a comparative analysis was performed before (pre-PC) and after (post-PC) the photocatalytic process. The analysis was performed for all samples and is shown in the SI (Figures S6­(a–h), S7­(a–f), and S8­(a–g)).

### 3.4 SMX Degradation

Photocatalytic degradation tests of SMX under visible light revealed a strong dependence on the synthesis medium of ASO microcrystals, which modulated both the material’s defect chemistry and structural order. The data on photocatalysis efficiency and kinetics (Figure 5(a–b)) demonstrate that ASOet exhibited the highest photocatalytic performance, with a first-order rate constant of 4.94 × 10^–3^ min^–1^ (*R* ^2^ = 0.997), surpassing ASOwat (2.31 × 10^–3^ min^–1^) and ASOamm (1.70 × 10^–3^ min^–1^). This superior activity is attributed to the optimized morphological features of ASOet (highly faceted, uniform rods with minimal deep-level defects) as well as its predominant red PL emission and intensified Se–O vibrational modes, indicating favorable charge separation and surface activation. Additionally, sample ASOet showed the best mineralization behavior (∼54%), compared to samples ASOwat (no significant mineralization) and ASOamm (∼33%).

**Figure 5:**
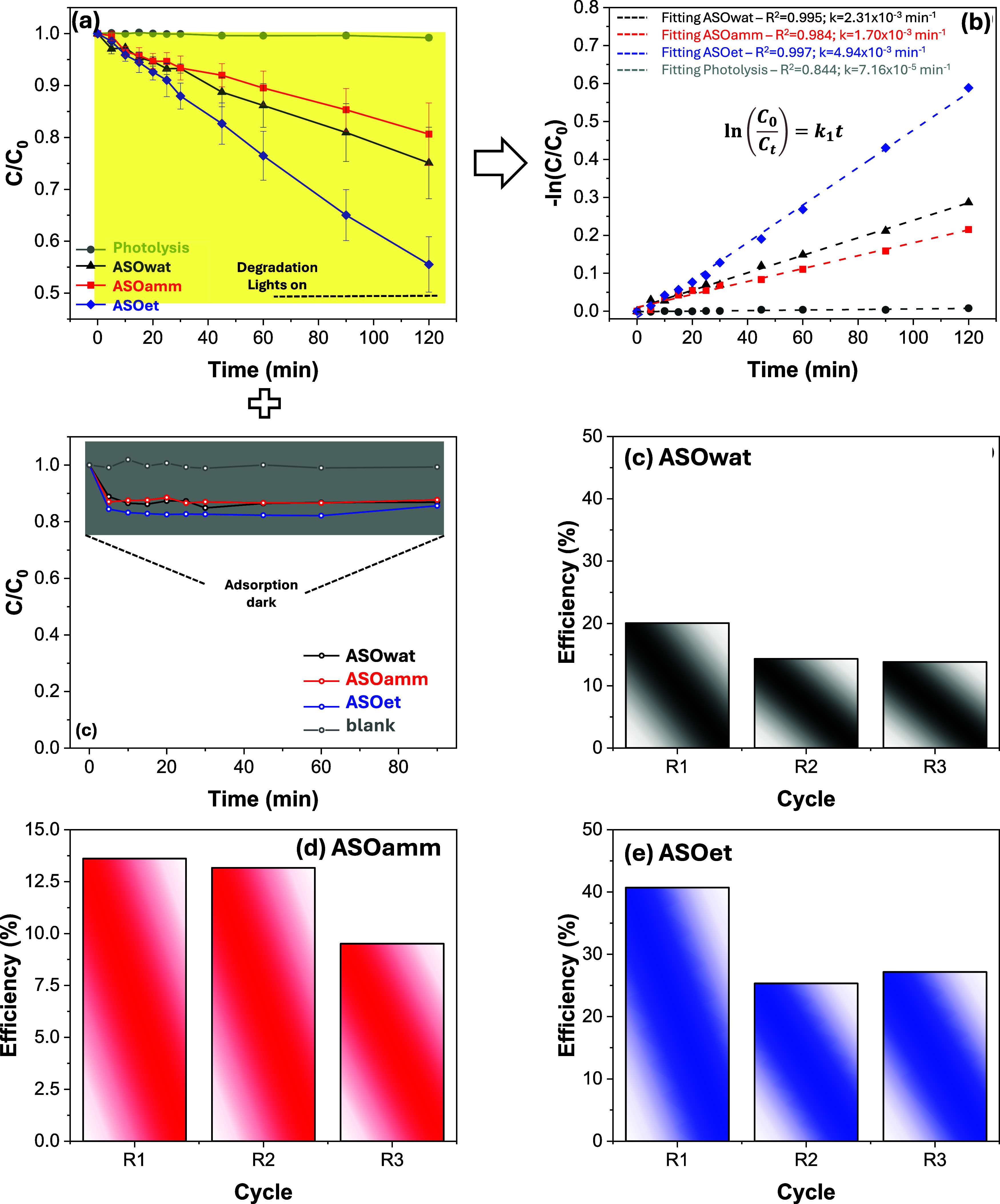
Photocatalytic (a) performance with an inset showing adsorption dynamics over time and the respective (b) kinetics for the spectra of SMX degradation, and recyclability obtained for the samples synthesized in (c) aqueous (ASOwat), (d) ammoniacal (ASOamm), and (e) ethanolic (ASOet) environments.

Recyclability tests (Figure 5(c–e)) showed that ASOet maintained the highest degradation efficiency over three cycles, highlighting the durability of its active sites, whereas ASOamm and ASOwat suffered more pronounced deactivation, likely due to the presence of structural defects that act as recombination centers or degrade the catalytic surface over time.

The scavenger tests (Figure 6(a–c)) show that for ASOet, the presence of ascorbic acid (^1^O_2_ scavenger) led to a complete suppression of SMX degradation, indicating a strong dependence on superoxide radicals. Additionally, the partial inhibition observed with potassium biftalate (^•^OH scavenger) suggests that the hydroxyl radical also plays a secondary but relevant role. Interestingly, the ASOwat and ASOet samples showed degradation strongly suppressed by ascorbic acid and potassium biftalate, implying that both superoxide and hydroxyl radicals are the main reactive species. Meanwhile, ASOamm showed the least sensitivity to individual scavengers, suggesting a less efficient or less specific photocatalytic pathway dominated by nonselective recombination. Across all samples, ammonium oxalate (h^+^ scavenger) caused only minor inhibition, indicating that the positively charged VB has a minimal influence. Instead, the degradation is dominated by electron-driven ROS, especially ^1^O_2_.^46,47^

**Figure 6:**
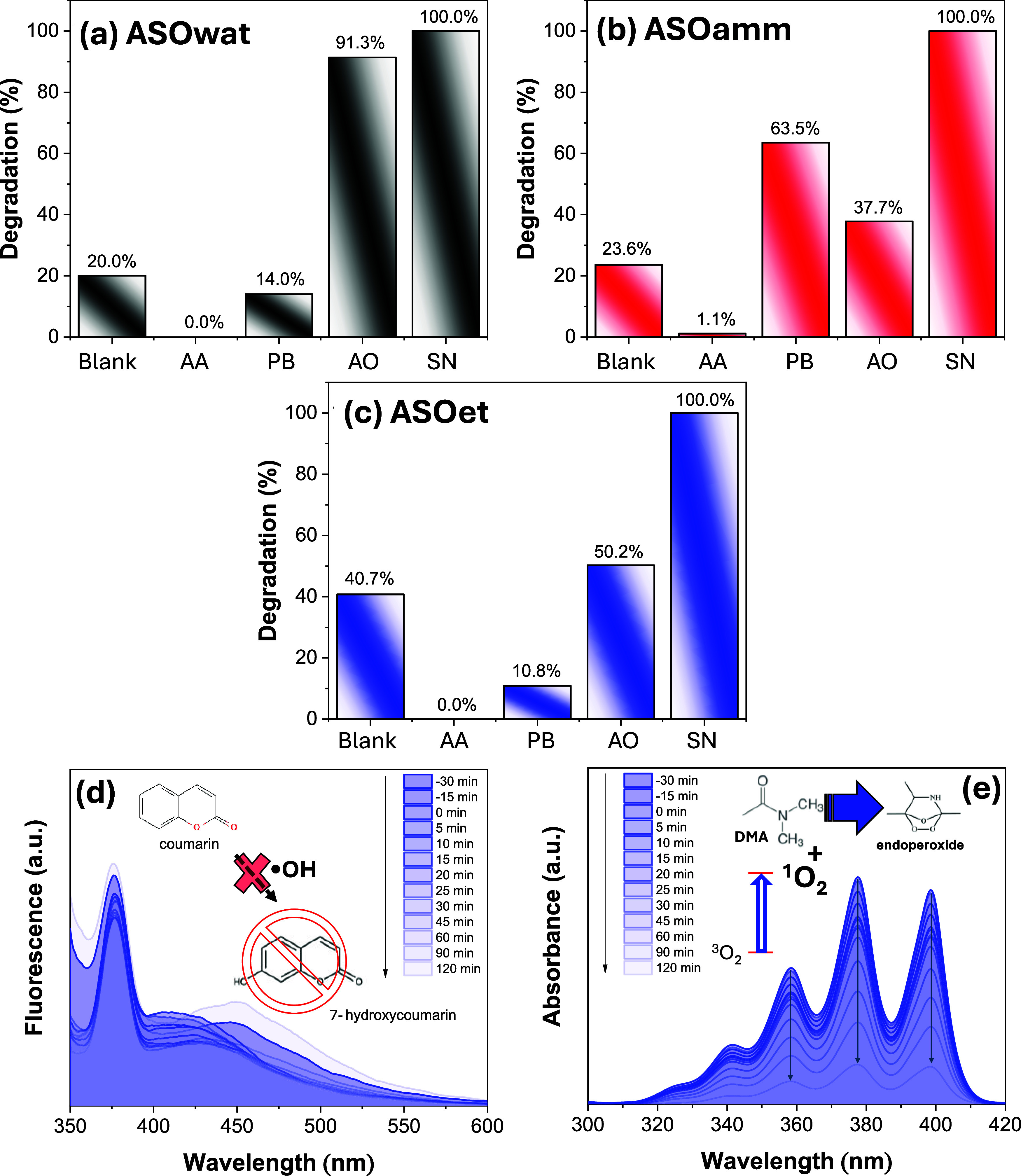
(a–c) Scavengers’ tests for the samples synthesized in aqueous (a) (ASOwat), (b) ammoniacal (ASOamm), and (c) ethanolic (ASOet) environments. Probing experiments using (d) coumarin (^•^OH) and (e) DMA (^1^O_2_) were performed for the sample ASOet. Scavengers used are AA = ascorbic acid, PB = potassium biftalate, AO = ammonium oxalate, and SN = silver nitrate.

These results support a degradation mechanism wherein, upon visible-light excitation, the ASO promotes electronic transitions from the valence band to the conduction band. In ASOet, these electrons efficiently reduce O_2_ to generate ^•^O_2_^–^ radicals, which can further react to form ^•^O_2_H and ^1^O_2_ species. On the other hand, surface hydroxyl groups or water are oxidized by the resulting holes or reactive intermediates to form ^•^OH radicals. Finally, photogenerated electrons are quickly scavenged by Ag^+^ to form metallic silver species (Ag^0^). By removing electrons from the CB, AgNO_3_ reduces electronic recombination, thereby increasing the charge separation lifetime and the positive state of the VB. This behavior suggests that charge-carrier separation is a limiting factor toward the degradation of SMX, which is improved for sample ASOet. These reactive oxygen species synergistically drive the oxidation of SMX. The reduced defect density and dominant red PL emission in ASOet, as shown previously, facilitate prolonged charge-carrier lifetimes and minimize recombination, enabling more efficient generation of ROS. In contrast, ASOwat and ASOamm, which contain higher densities of Ag and Se vacancies and broader PL emissions, exhibit higher electronic recombination rates, lower ROS yields, and consequently lower photocatalytic activity.

In addition to scavengers’ tests (indirect), probes can be used to directly identify the formation of these species (*e.g.*, ^1^O_2_, OH), detecting specific molecules formed exclusively in the presence of specific ROS. Coumarin, a nonfluorescent compound, undergoes a specific reaction in the presence of ^•^OH radicals, resulting in the formation of 7-hydroxycoumarin, a fluorescent species with a maximum emission between 400 and 500 nm, detectable through fluorescence spectrophotometry. Probing experiments were conducted for sample ASOet, which performed the best. Figure 6(d) presents the evolution of the fluorescence spectra obtained in the presence of both catalysts at reaction times of −30, −15, 0, 5, 10, 15, 20, 25, 30, 45, 60, 90, and 120 min. The peak of 7-hydroxycoumarin shows no increase in intensity, even after 120 min of reaction, indicating low formation of ^•^OH radicals in the presence of the ASO. Considering the scavenging tests, another possible explanation for the probe experiments is that ^•^OH radicals may be generated at such a fast rate that they rapidly react to form ^•^O_2_H or the singlet oxygen, maximizing the reaction, even though it cannot be detected in these experiments. Analogously, DMA was used to evaluate ^1^O_2_ species’ formation (Figure 6(e)). Upon reaction with ^1^O_2_, endoperoxide is formed, reducing the intensity associated with DMA absorption. Thus, it signals ^1^O_2_ production by the photocatalyst. The results show a steep decrease in DMA absorption over 120 min, which indicates the intense generation of ^1^O_2_ species as a result of the interaction between ASO and the medium. Therefore, it can be inferred that ^1^O_2_ radicals are also involved in the sulfide oxidation mechanism.

### 3.5 Mechanistic Pathways and Closing Remarks

This work provides conclusive proof, combining experimental and theoretical data, of how the synthesis medium can be used to tailor the photocatalytic response of ASO-based semiconductors. According to the results, it is particle morphology that modulates the final properties of the semiconductor. This can be ascribed to the different energy and defect concentration pattern observed, which can be associated with the exposed planes, and also to the kind and charge of the silver ([AgO_*x*_]) and selenium (SeO_3_) quantum clusters. The exposed surface array modulates the interaction between the semiconductor and the environment in which it is exposed, regulating electronic dynamics. Negatively charged surfaces improve electron donation, whereas positively charged surfaces favor electron acceptance, which in turn affects the ROS generation profile. Corroborating the experimental and theoretical data further supports these findings. For instance, the effective surface *E*_gap_ changes as a function of the exposed surfaces. These differences are significant and will directly influence the reactivity of the semiconductor with its environment and, thus, its ability to generate ROS.

Apart from structural and electronic defects, the morphology of a semiconductor (i.e., ASO) is deterministic of its properties. Each exposed surface in a semiconductor accounts for a different energetic profile, with a unique *E*_gap_ defined by the presence of different silver and selenium quantum cluster arrays. In the sample ASOet, the surface (101) represents a higher percentage compared to samples ASOwat and ASOamm. Although small, this difference produces significant changes in the reactivity of the sample, enhancing the photocatalytic response of that sample. Apart from the surface (101), the presence of the low-reactivity surfaces, (010) and (100), hinders the photocatalytic response. Figure 7 shows a schematic representation of the proposed mechanism ascribed to the photodegradation of SMX molecules in an aqueous medium in terms of the morphology and its influences on ROS generation.

**Figure 7:**
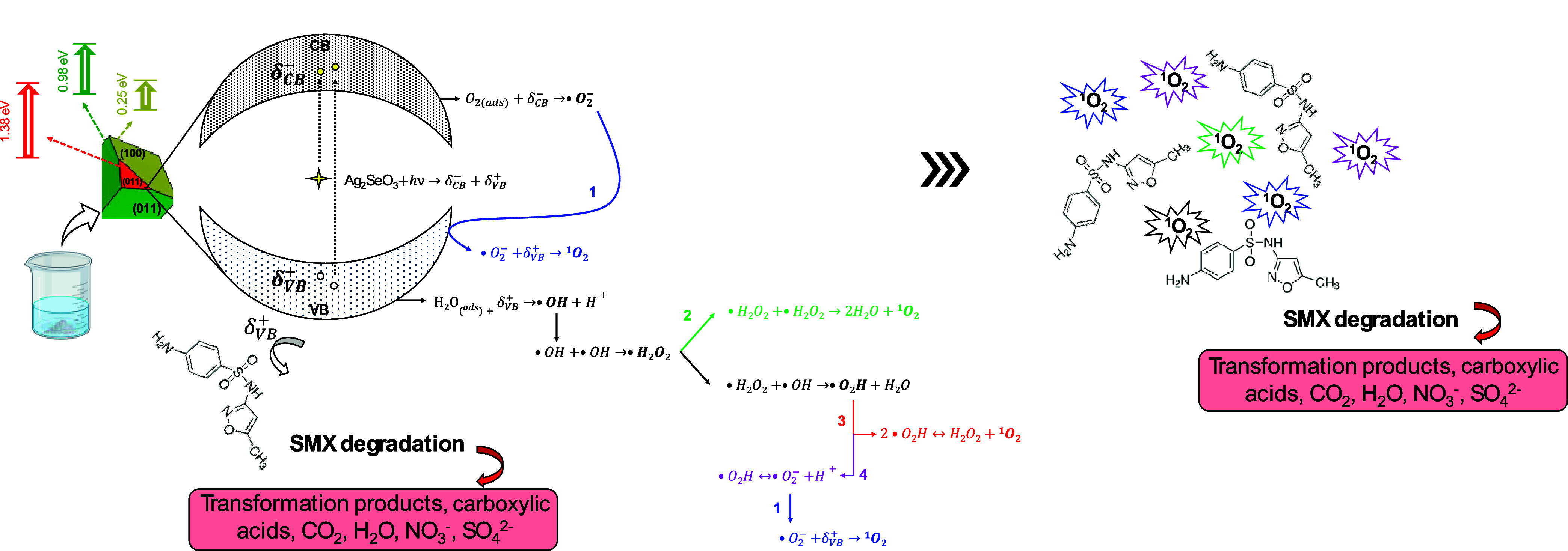
Mechanistic pathways describing the photodegradation of SMX molecules in an aqueous medium.

When the semiconductor is irradiated by a visible-light source, the electrons are promoted from intermediary energy levels within the band gap region and the VB to the CB, generating charge separation (eqs 1a). In an aqueous solution, O_2(g)_ is reduced at the positively charged region (δ_CB_^–^) to form ^•^O_2_^–^, and subsequently the ^1^O_2_ (eqs 3–4), whereas hydrolysis produces ^•^OH radicals and H^+^ at the positive region (δ_VB_^+^) according to (eqs 5).

2 $$\mathrm{ASO}+h\nu \to {\delta_{\mathrm{CB}}}^{-}+{\delta_{\mathrm{VB}}}^{+}$$

3 O2(ads)+δCB−→O2−•

4 O2−•+δVB+→O21

5 H2O(ads)+δVB+→OH•+H+

The results also indicate that the ^•^OH radicals, generated at an accelerated rate, quickly follow cascade reactions to form H_2_O_2_ and the ^•^O_2_H radical, which will ultimately form the ^1^O_2_ following eqs 6–11

6 OH•+OH•→H2O2•

7 H2O2•+H2O2•→2H2O+O21

8 H2O2•+OH•→O2H•+H2O

9 2O2H•↔H2O2+O21

10 O2H•↔O2−•+H

11 O2−•+δVB+→O21

Following ROS generation, SMX molecules are then degraded to form transformation products, carboxylic acids, CO_2_, H_2_O, NO_3_^–^, and SO_4_^2–^. Furthermore, Ag_2_SeO_3_ (δ_VB_^+^) may also attack SMX molecules directly.

Table 3 shows a comparison between the ASO obtained in this work and other ASO-based materials applied in photocatalysis. Studies regarding the photocatalytic response of ASO semiconductors are still scarce, especially considering the photodegradation of pharmaceutical pollutants and using a visible-light source. Moreover, the available literature only shows the degradation efficiency and kinetics. This work uses TOC removal analysis to evaluate the real removal efficiency associated with the photocatalytic degradation of SMX. The TOC data suggests that the majority of SMX is mineralized; hence, no organic byproducts are left over in the solution.

**Table 3:** Degradation Efficiency Comparison with the Literature

photocatalyst	synthesis	source	pollutant	[C]_org_ (ppm)	[C]_cat_ (mg.ml^–1^)	Time (min)	*k* (min^–1^)	η (%)	TOC reduction (%)	refs
Ag_2_SeO_3_	SC (ethanolic médium, pH = 2)	Vis	SMX	1.00	1.00	120	4.94 × 10^**–**3^	∼55	∼54.0	this work
Ag_2_SeO_3_	SC (pH = 5)	UV	RhB	4.79	1.00	45		∼99	-	^26^
Ag_2_SeO_3_	SC, UT, MAH, CP	UV	RhB	4.79	1.00	60	5.51 × 10^**–**2^	∼100	-	^24^
PEDOT/Ag_2_SeO_3_	LLIS	IL	RhB - MB	1.00	0.50	210		∼23–∼46	-	^42^
Ag_2_SeO_3_/Ag_3_PO_4_/MWCNT/PVDF	CP	Vis	IC	10.0	0.03	50	4.53 × 10^**–**2^	∼90	-	^43^

## 4 Conclusions

This work demonstrates how the synthesis medium can be used to modulate morphology and tune the photocatalytic response of ASO synthesized via a sonochemical method. Pure-phase monoclinic ASO was obtained and successfully applied in the degradation of SMX under visible-light irradiation. Our results indicated a higher performance for the ASO sample synthesized in an ethanolic environment (water: ethanol (90–10) mixture), achieving ∼55% SMX degradation and 54% TOC mineralization within 120 min.

The experimental data were further corroborated by Wulff constructions and surface-energy calculations. The results show that the photocatalytic response of ASO-based materials is closely related to the structure of the material, considering the formation of shallow point defects such as oxygen vacancies and more energetic structural defects. In particular, the presence of the (101) surface in ASOet improves reactivity in the degradation environment, resulting in a more effective ROS generation and, thus, enhanced photocatalytic efficiency, as confirmed by the theoretical analysis. Conversely, low-reactivity surfaces can attenuate the activity of highly reactive surfaces, impairing the final response.

Importantly, the DOS and Mulliken charge analyses further reveal that oxygen vacancies reduce the degeneracy of the surface states and create localized defect levels near the CB, mainly associated with [SeO_3_] clusters. This electronic redistribution enhances charge separation and contributes to the observed photocatalytic behavior. In this sense, the synergy between morphology and local electronic structure determines the balance between active and inactive surfaces.

Apart from structural and electronic defects, this work provides conclusive evidence that particle morphology is deterministic of the final properties of ASO samples. Moreover, a step-by-step synthesis method to vary the material properties is provided. Each exposed surface in a semiconductor corresponds to a different energetic profile, with a unique band gap energy defined by the presence of different quantum cluster arrays. Thus, our work reinforces the critical importance of particle morphology: the individual band gaps of exposed surfaces supersede the overall band gap energy of the material, making visible-light excitation possible even for larger band gap materials.

## Supplementary Material



## Notes

Additional experimental details, results of phase purity analysis (XRD-Rietveld), absorbance spectra, band structure, density of states, XPS spectra, and photocatalyst analysis postphotocatalysis (PDF)
