# Supplementary material for: Understanding the Role of Morphology in the Visible-Light-Driven Sulfamethoxazole Degradation by Ag2SeO3‑Based Photocatalysts Synthesized in Different Solvent Media: An Experimental–Theoretical Approach
Source: Inorg Chem. 2026 Jan 21;65(4):2269–83. doi: 10.1021/acs.inorgchem.5c04860 (PMC12869502; doi:10.1021/acs.inorgchem.5c04860)
Supplement: Supplementary file 1 [file ic5c04860_si_001.pdf]

Supporting Information

Understanding the role of morphology in the visible light driven sulfamethoxazole degradation by Ag_2SeO_3-based photocatalysts synthesized in different solvent media: An experimental-theoretical approach

Henrique Moreno[a*], Laura O. Libero[a], Amanda Fernandes Gouveia[b], Marcio Daldin Teodoro[c], Monica Calatayud[b], Alexandre Zirpoli Simões[d], Elson Longo[a].

[a] Center for Research and Development of Functional Materials, Federal University of São Carlos (UFSCar), 13565-905, São Carlos, Brazil.
[b] Sorbonne Université, CNRS, MONARIS, CNRS-UMR 8233, 4 Place Jussieu F-75005 Paris, France
[c] Department of Physics, Federal University of São Carlos, São Carlos, SP, 13565-905, Brazil.
[d] Faculty of Engineering of Guaratinguetá, São Paulo State University, 12516-410, Guaratinguetá, SP, Brazil.

Corresponding author: hmoreno@ufscar.br

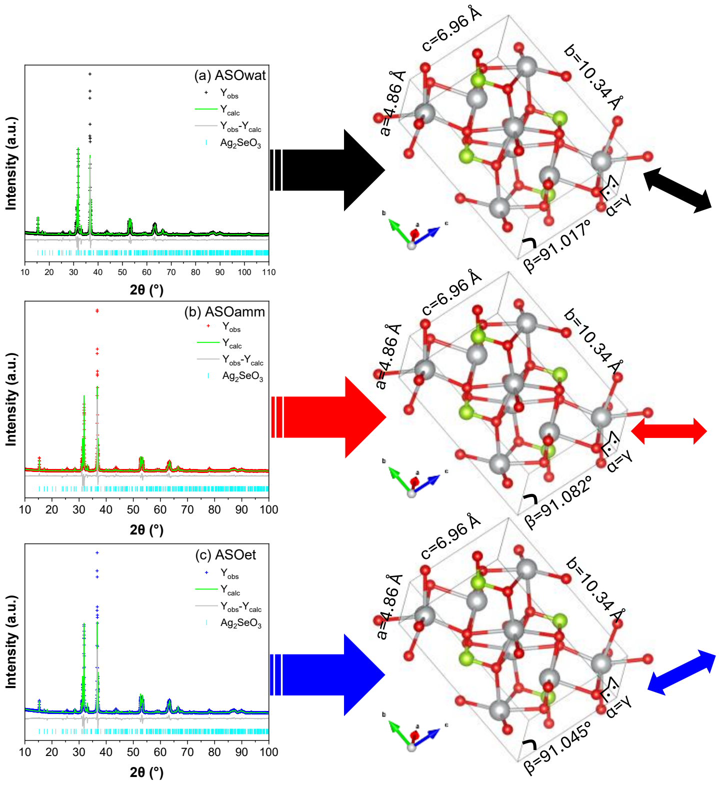

Sample	Atom	X	Y	Z
ASOwat	Ag1	0.26346	0.24497	0.08541
	Ag2	0.26448	0.59471	0.62606
	Se1	0.72688	0.41433	0.83391
	O1	0.38989	0.40815	0.87952
	O2	0.80662	0.57062	0.62187
	O3	0.88311	0.26084	0.59712
ASOamm	Ag1	0.25372	0.24758	0.09051
	Ag2	0.27731	0.59087	0.62361
	Se1	0.75220	0.41722	0.84166
	O1	0.39005	0.42486	0.87715
	O2	0.90462	0.56256	0.62053
	O3	0.83107	0.24413	0.60053
ASOet	Ag1	0.26385	0.25134	0.08763
	Ag2	0.26134	0.57992	0.63081
	Se1	0.73165	0.40760	0.83412
	O1	0.43204	0.40364	0.87770
	O2	0.80784	0.57594	0.63810
	O3	0.84797	0.24903	0.60802

Figure S1 – XRD-Rietveld analysis for the samples synthesized in (a) aqueous (ASOwat), (b) ammoniacal (ASOamm), and (c) ethanolic (ASOet) environments. The respective lattice parameters (a, b, c, α, β, γ) and atomic positions (x, y, z) are shown as inserts.

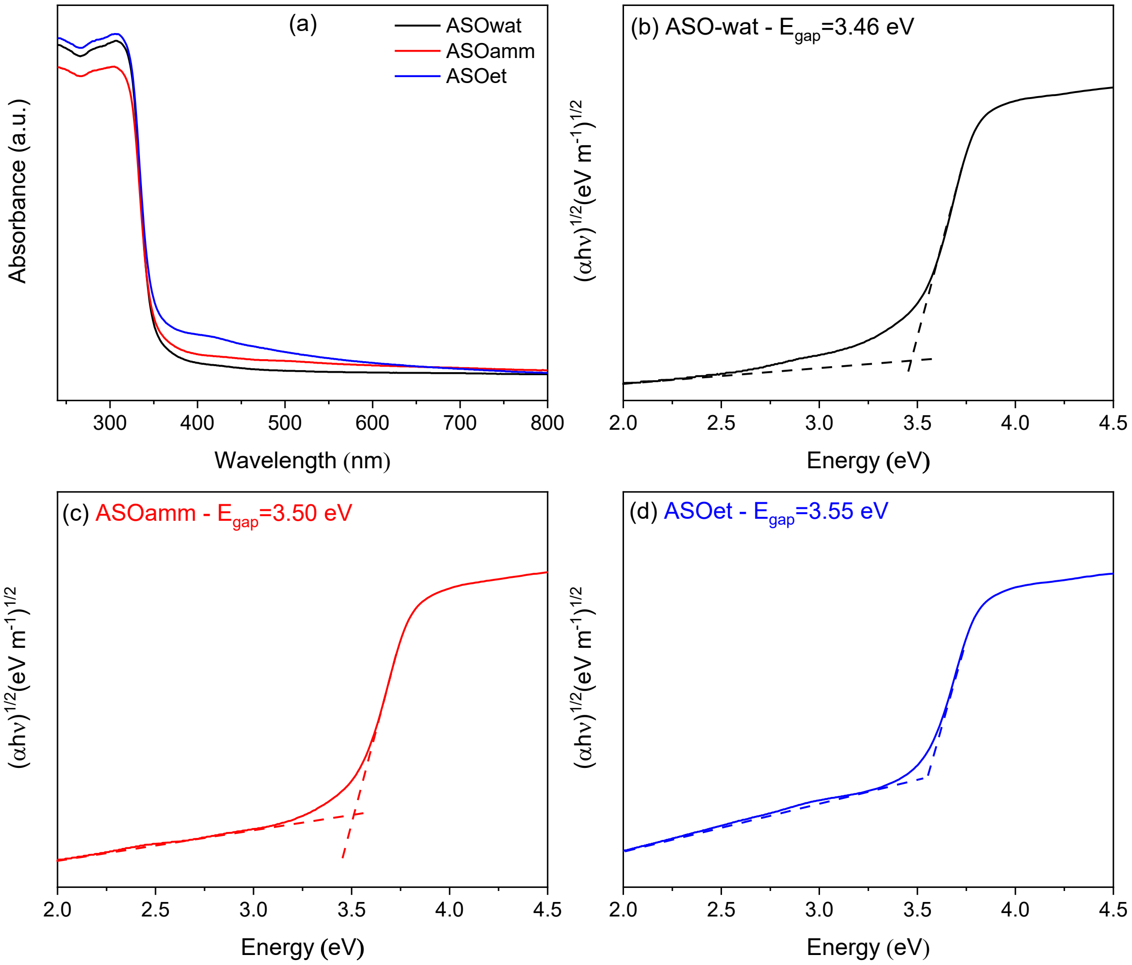

Figure S2 – (a) Absorbance spectra and the respective Tauc plots obtained for samples (b) ASOwat, (c) ASOamm and (c) ASOet.

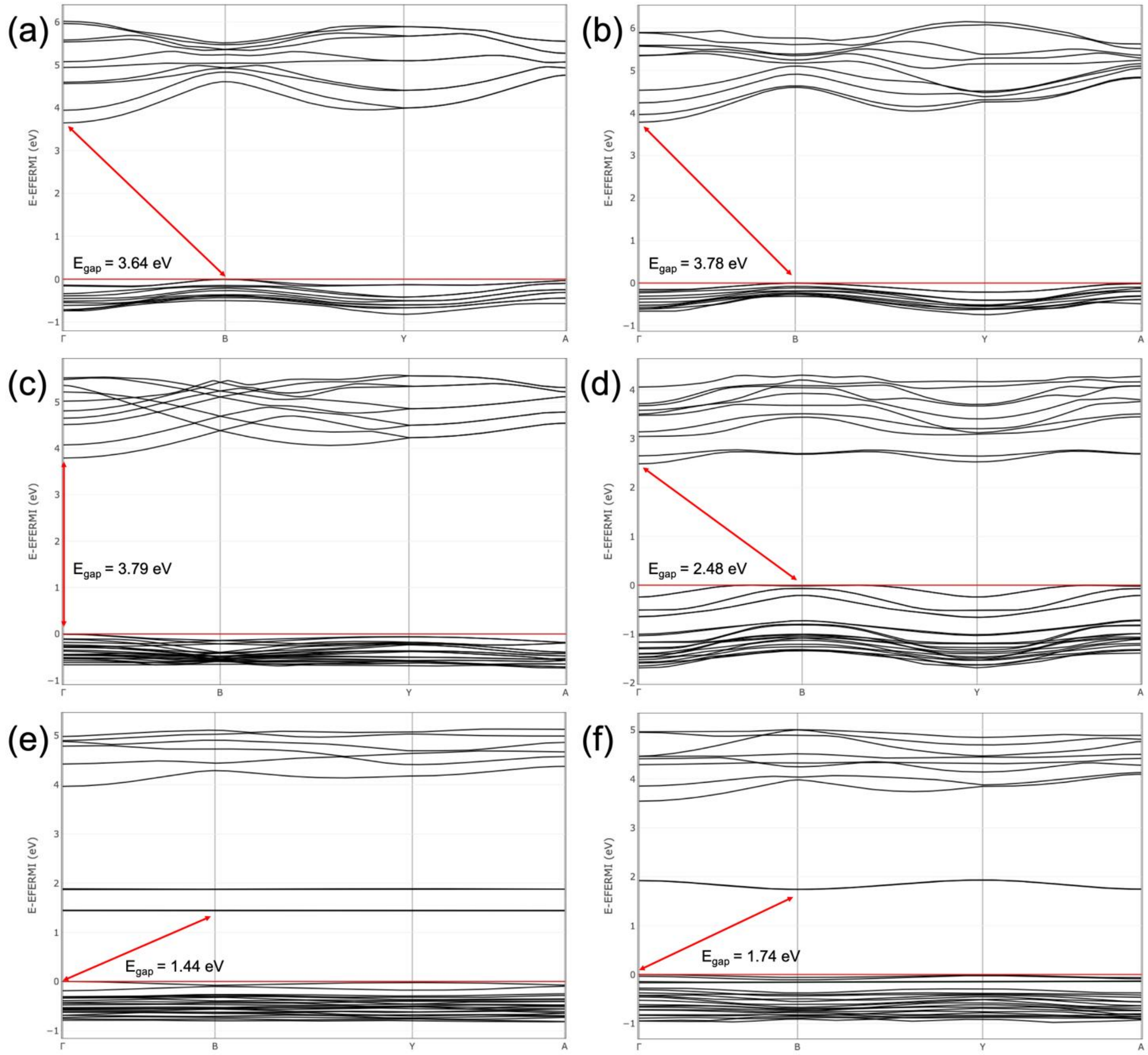

Figure S3 – Band structure of the (a) (001), (b) (010), (c) (100), (d) (011), (e) (101), and (f) (110) surfaces.

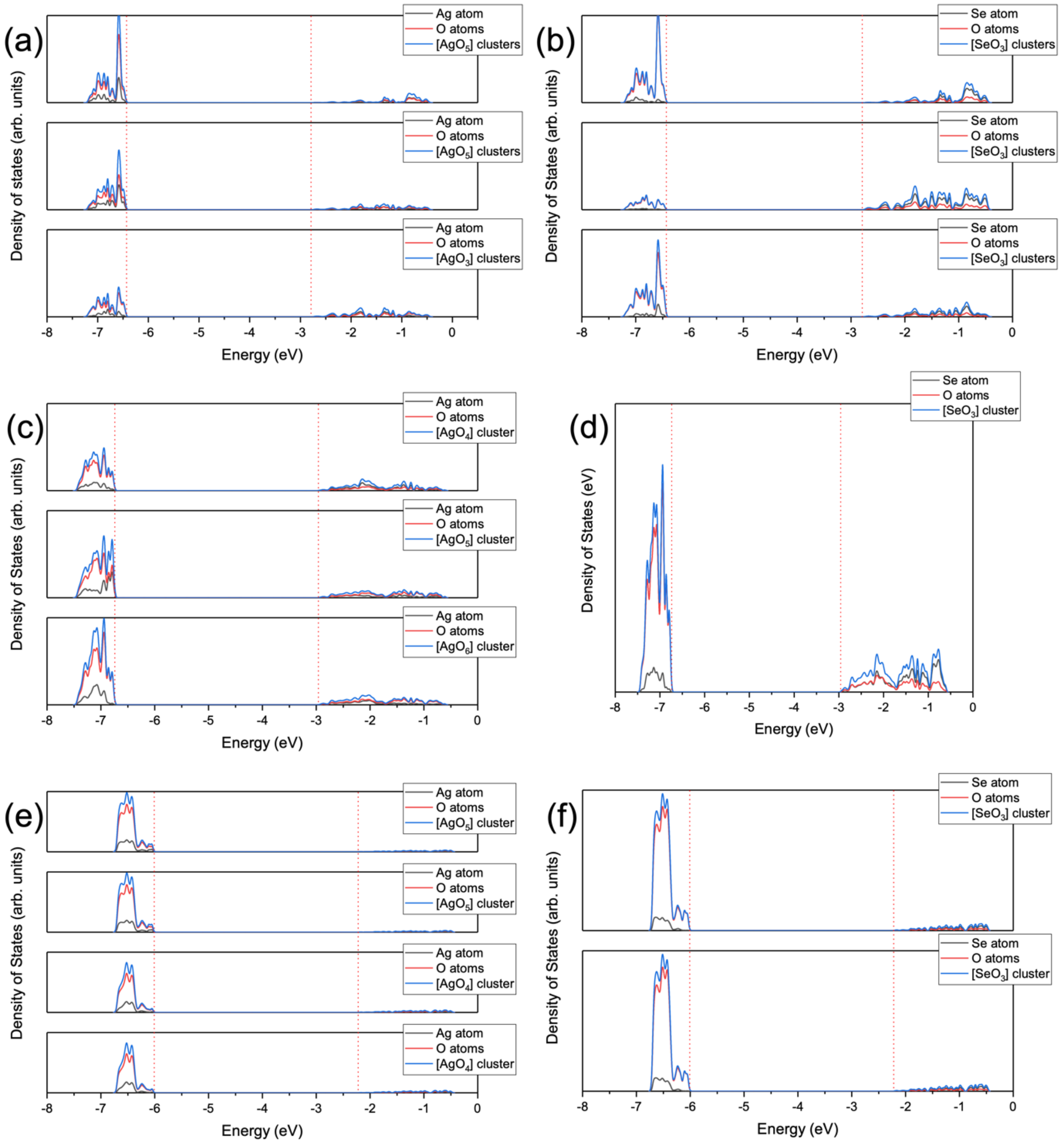

Figure S4 – Projected density of states for the [AgO_x] clusters and [SeO_x] clusters on the (a, b) (001), (c, d) (010), and (e, f) (100) surfaces.

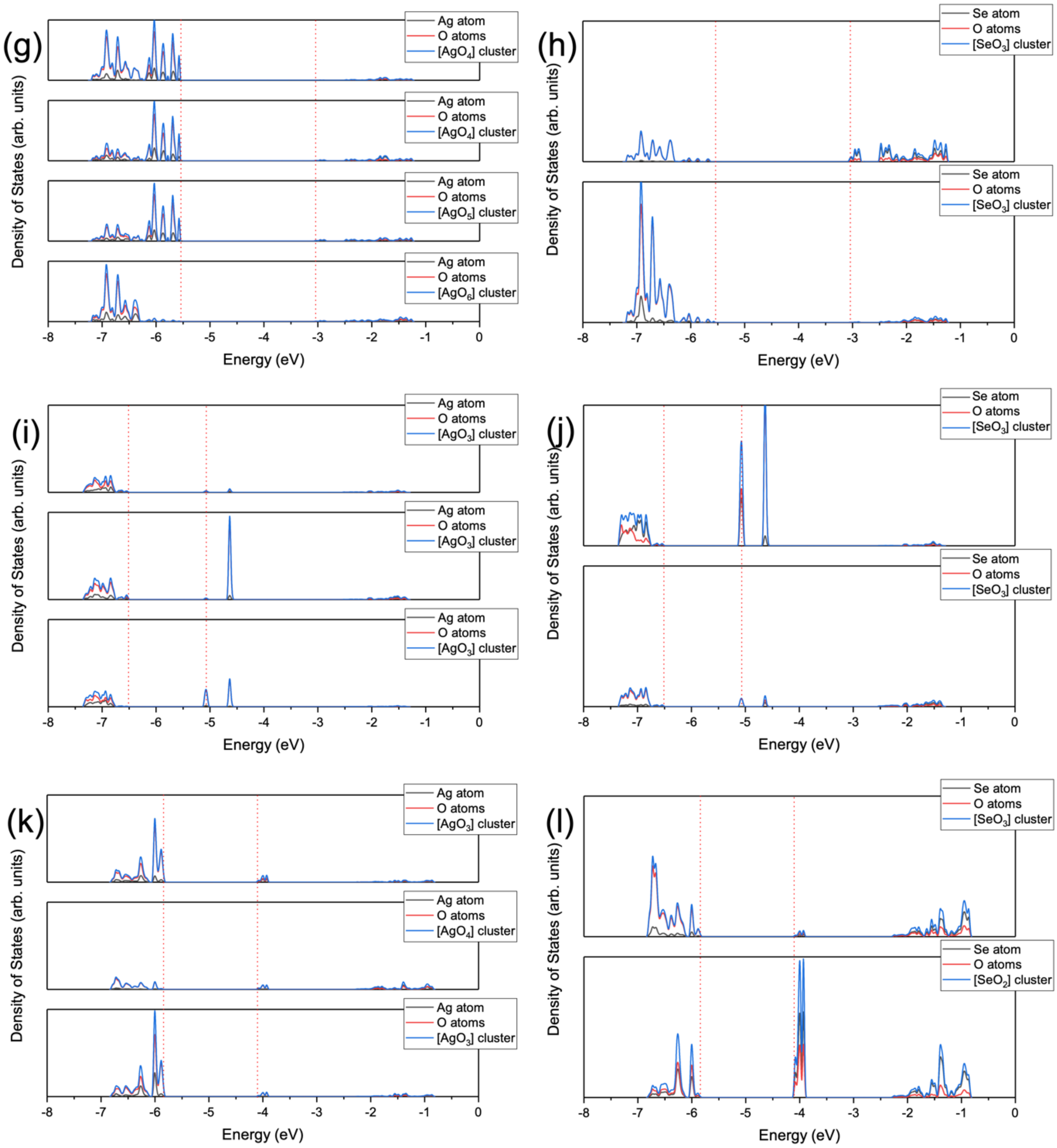

Figure S4 (continued) – Projected density of states for the $[AgO_x]$ clusters and $[SeO_x]$ clusters on the (g, h) (011), (i, j) (101), and (k, l) (110) surfaces.

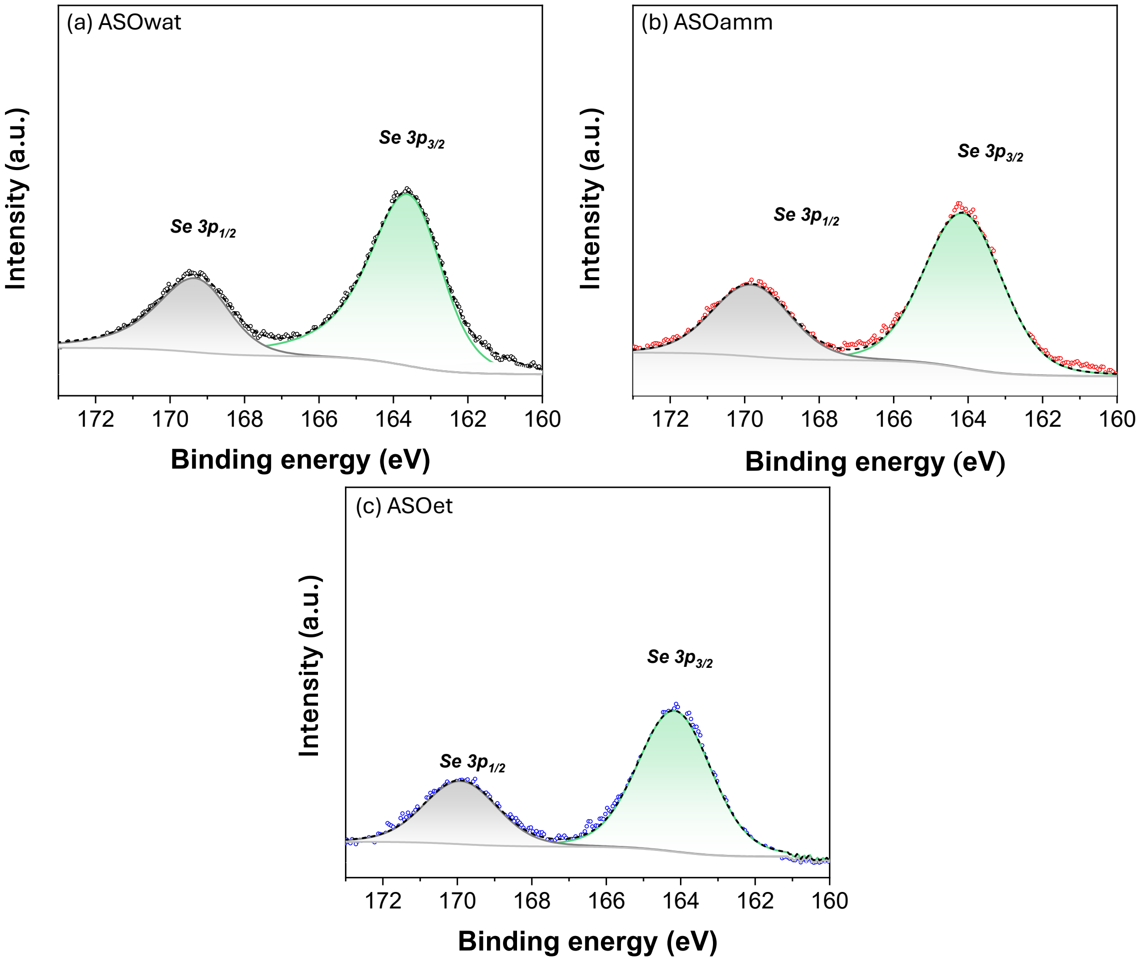

Figure S5 – Se 3p high-resolution XPS spectra obtained for the samples synthesized in (a) aqueous (ASOwat), (b) ammoniacal (ASOamm) and (c) ethanolic (ASOet) environment

<u>Photocatalyst analysis post-photocatalysis of Sulfamethoxazole</u>

To evaluate the structural integrity and the morphology of the ASO particles under visible light driven SMX degradation, a comparative analysis before (pre-PC) and after (post-PC) the photocatalytic process was performed for all samples.

Figure S6(a-d) and Figure S6(e-h) display the Raman and FTIR spectra for each of the samples before and after the photocatalytic degradation of SMX. Raman spectra after photocatalysis (dashed lines) show that the main vibrational mode at ~757 cm^{-1} (A_g) is still prominent in all samples, confirming retention of the SeO_3^{2-} cluster structure. However, band broadening and slight redshifts are observed in ASOwat and ASOamm, particularly in the region between 667–700 cm^{-1}, indicating an increase in structural disorder and local symmetry disruption due to accumulated defects such as oxygen or silver vacancies. FTIR spectra support this interpretation: the Se–O bending (~667 cm^{-1}) and stretching (~441 cm^{-1}) modes are preserved in ASOet, and significantly

increase in intensity for ASOwat and ASOamm after degradation. Additionally, the broad N–H/C–H band around 2990 cm^{-1} remains relatively stable in ASOet and ASOamm, indicating limited volatilization or surface loss of organic residues.

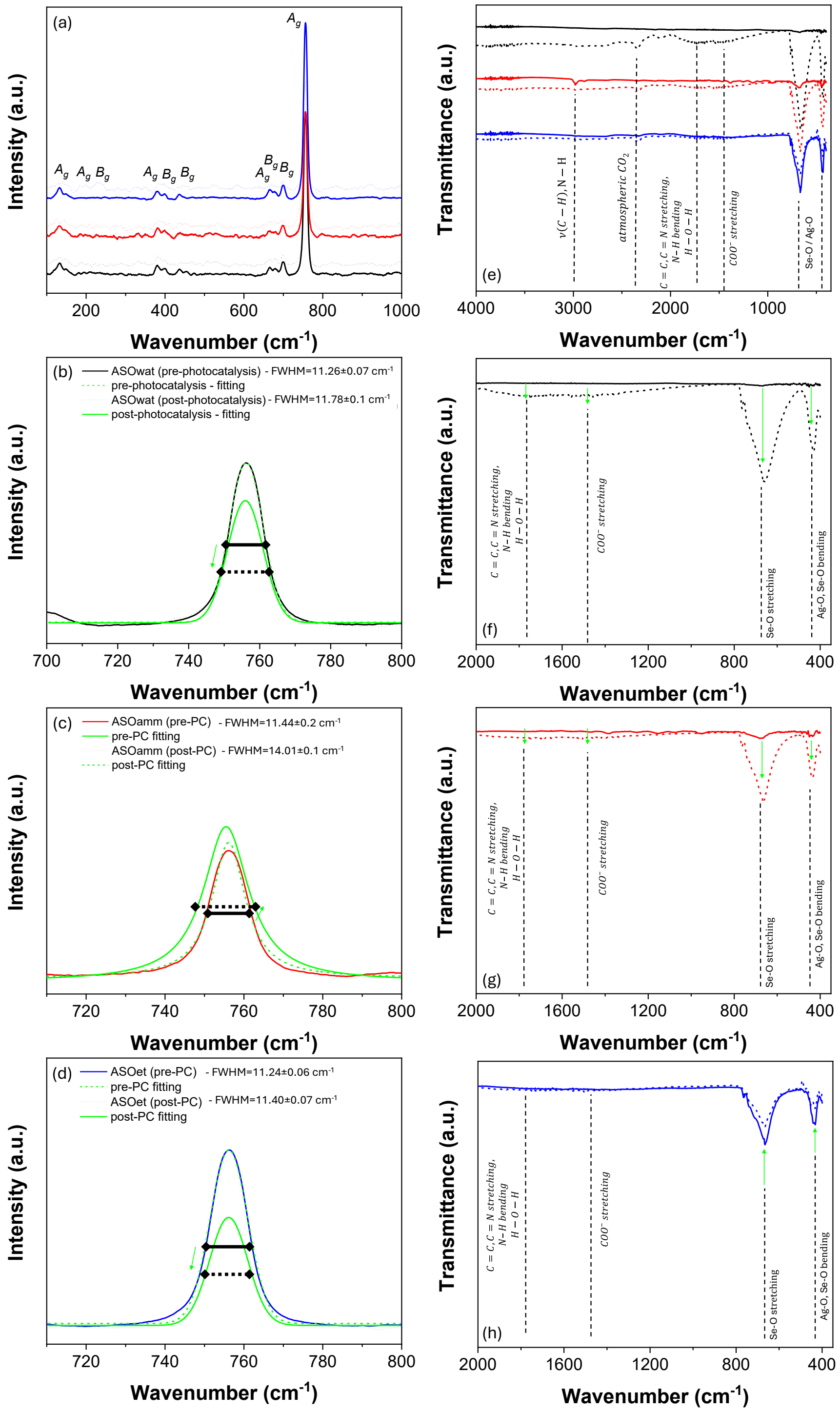

Figure S6 – (a) Raman spectra and (b-d) fitting of the main vibrational (A_g, at ~757 cm^{-1}) showing FWHM values for each of the samples synthesized in aqueous (ASOwat, black), ammoniacal (ASOamm, red), and ethanolic (ASOet, blue) environments. (e) FTIR spectra and (f-h) the

respective zoomed in spectrum (between 2000 and 350 cm^{-1}) for each of the samples ASOwat, ASOamm, and ASOet.

FE-SEM micrograph images (**Figure S7(a-c)**) obtained after (post-PC) the photocatalytic process shows that while all samples retain their rod-like morphology, clear morphological degradation occurs in ASOwat and ASOamm, including surface roughening, particle fragmentation, and collapse of smaller rods attached to larger ones. In contrast, ASOet maintains a more defined morphology with minimal surface etching, supporting its superior resistance to photocorrosion and oxidative degradation.

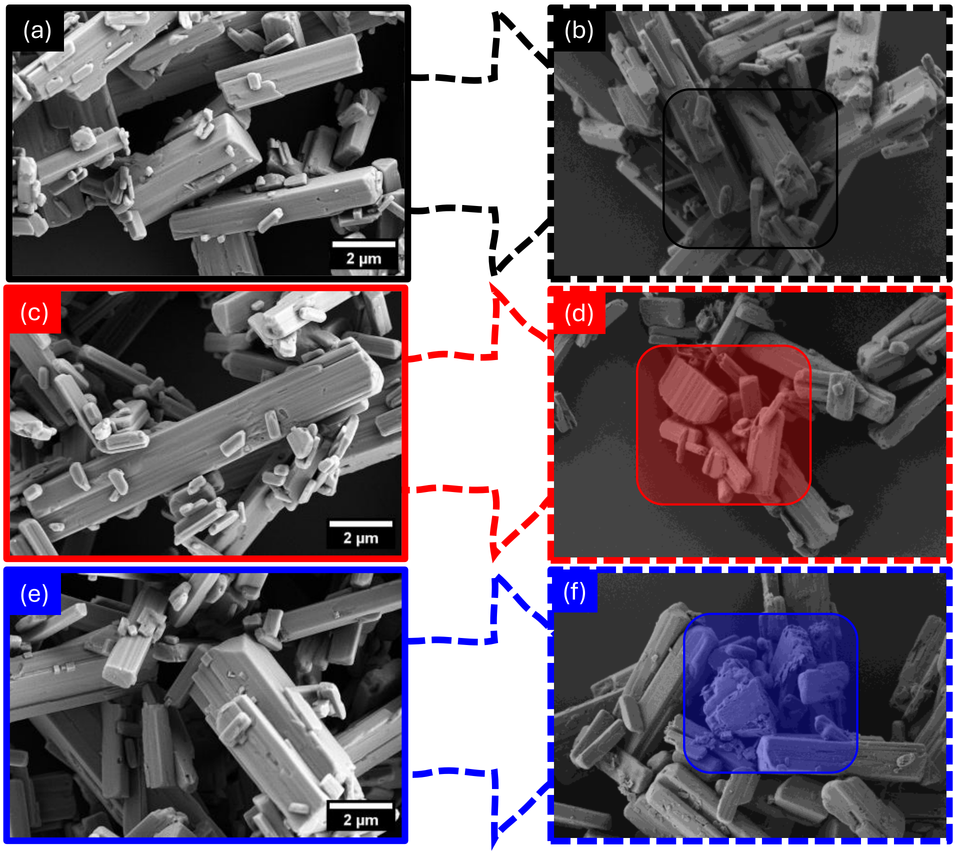

Figure S7 – FE-SEM micrograph images showing the evolution of the morphology of the samples (a, c, e) before and (b, d, f) after the photocatalytic degradation of SMX for samples (a-b) ASOwat, (c-d) ASOamm, and (e-f) ASOet.

Photoluminescence data pre-PC and post-PC (**Figure S8(a-g)**) provide further evidence of evolving defect states. For ASOwat and ASOet, PL intensity increases after degradation (post-PC, dashed lines) compared to the samples pre-PC (filled lines), particularly in the NIR and deep-red regions, which may be associated with the formation of deep-level traps (*e.g.,* Ag and Se vacancies). These states are known to promote non-radiative recombination and lower catalytic efficiency upon reuse. After photocatalysis (post-PC) deconvolution of the PL spectra shows violet

emission peaks (~2.90 eV). This indicates a significant blue-shift in the emission of the samples post-PC compared to that of the samples pre-PC, which indicates that the photocatalytic process stabilizes the ASO lattice, generating oxygen vacancies in exchange.

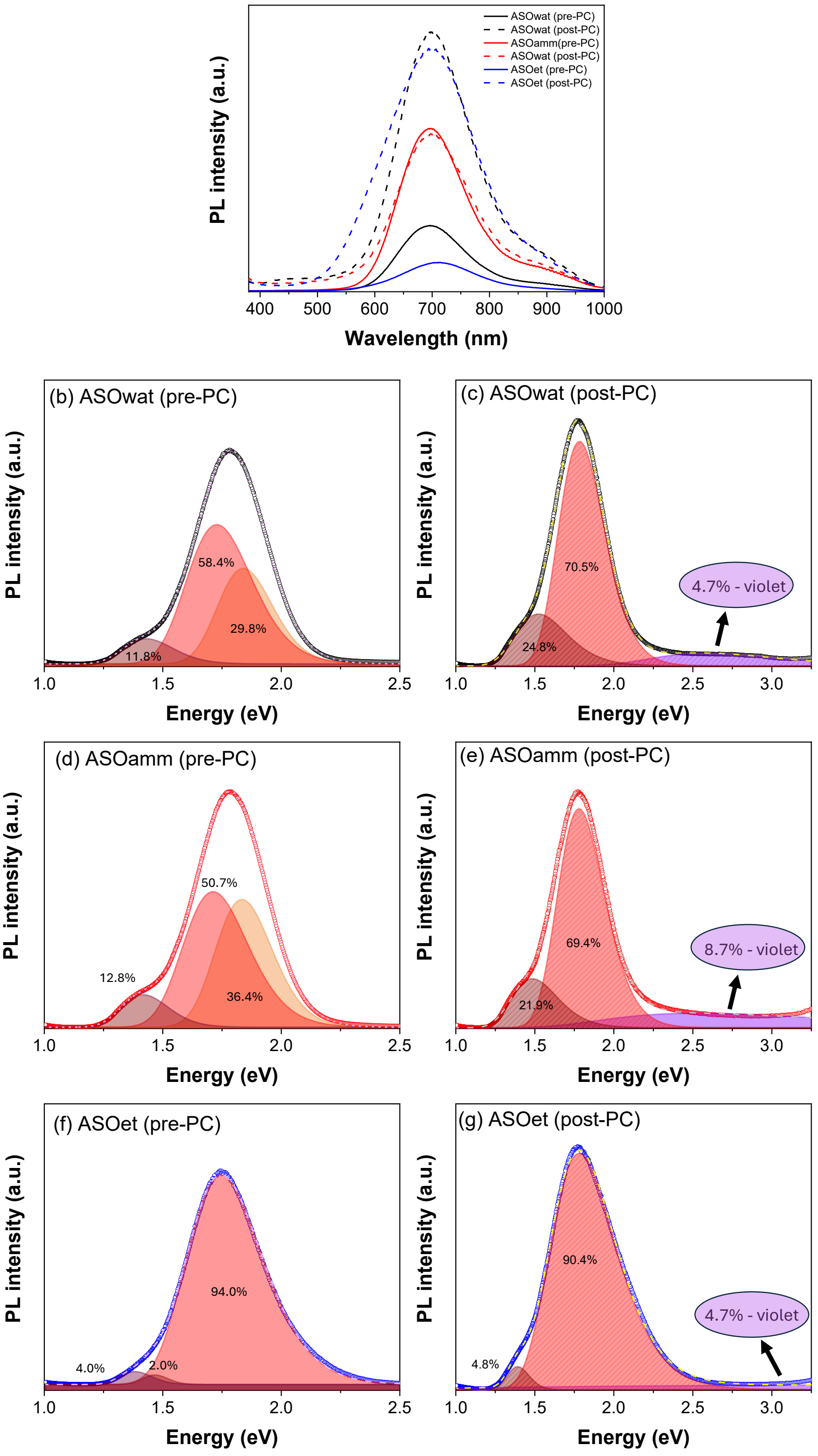

Figure S8 – (a) PL spectroscopy measurements and deconvolution showing the evolution of the emission behavior of the samples (b, d, f) before and (c, e, g) after the photocatalytic degradation of SMX for samples (b-c) ASOwat, (d-e) ASOamm, and (f-g) ASOet.

Table S1 – Lattice and Rietveld refinement parameters for all ASO samples synthesized in (a) aqueous (ASOwat), ammoniacal (ASOamm), and ethanolic (ASOet) environments.

Sample	Volume (Å^3)	a (Å)	b (Å)	c (Å)	α=γ (°)	β (°)	R_{wp}	GoF
ASOwat	349.5	4.86	10.34	6.96	90	91.017	14.4	4.8
ASOamm	349.2	4.86	10.34	6.95	90	91.082	16.4	5.5
ASOet	349.4	4.86	10.34	6.95	90	91.045	12.3	3.8
